# Sustained mitogen-activated protein kinase activation reprograms defense metabolism and phosphoprotein profile in *Arabidopsis thaliana*

**DOI:** 10.3389/fpls.2014.00554

**Published:** 2014-10-20

**Authors:** Ines Lassowskat, Christoph Böttcher, Lennart Eschen-Lippold, Dierk Scheel, Justin Lee

**Affiliations:** ^1^Department of Stress and Developmental Biology, Leibniz Institute of Plant BiochemistryHalle/Saale, Germany; ^2^Federal Research Centre for Cultivated Plants, Institute for Ecological Chemistry, Plant Analysis and Stored Product Protection, Julius Kühn InstituteBerlin, Germany

**Keywords:** MAPK substrates, defense, phosphoproteomics, metabolomics, phytoalexins, phosphorylation

## Abstract

Mitogen-activated protein kinases (MAPKs) target a variety of protein substrates to regulate cellular signaling processes in eukaryotes. In plants, the number of identified MAPK substrates that control plant defense responses is still limited. Here, we generated transgenic *Arabidopsis thaliana* plants with an inducible system to simulate *in vivo* activation of two stress-activated MAPKs, MPK3, and MPK6. Metabolome analysis revealed that this artificial MPK3/6 activation (without any exposure to pathogens or other stresses) is sufficient to drive the production of major defense-related metabolites, including various camalexin, indole glucosinolate and agmatine derivatives. An accompanying (phospho)proteome analysis led to detection of hundreds of potential phosphoproteins downstream of MPK3/6 activation. Besides known MAPK substrates, many candidates on this list possess typical MAPK-targeted phosphosites and in many cases, the corresponding phosphopeptides were detected by mass spectrometry. Notably, several of these putative phosphoproteins have been reported to be associated with the biosynthesis of antimicrobial defense substances (e.g., WRKY transcription factors and proteins encoded by the genes from the “*PEN*” pathway required for penetration resistance to filamentous pathogens). Thus, this work provides an inventory of candidate phosphoproteins, including putative direct MAPK substrates, for future analysis of MAPK-mediated defense control. (Proteomics data are available with the identifier PXD001252 *via* ProteomeXchange, http://proteomecentral.proteomexchange.org).

## Introduction

As sessile organisms, plants rely predominantly on physical barriers and adaptive mechanisms to adapt to biotic and abiotic stress conditions. During infection, pathogens can trigger PTI (pattern-triggered immunity) after recognition of conserved microbe-associated molecular patterns (MAMPs) or damage-associated molecular patterns (DAMPs), which are plant endogenous components released during tissue wounding, e.g., from herbivores or lytic enzymes released by pathogens. Binding of these MAMP/DAMP ligands to surface exposed pattern recognition receptors (PRRs) leads to activation of a variety of defense reactions to counteract the negative impact of the stresses (Boller and Felix, 2009; Knogge et al., 2009; Nicaise et al., 2009; Zipfel, 2009; Ranf et al., 2011).

The signaling network after MAMP/DAMP recognition includes ion fluxes (including a rise in cytosolic calcium concentrations), accumulation of reactive oxygen species and various phosphorylation events (Knogge et al., 2009; Liese and Romeis, 2013). Phosphorylation events appear to be key regulatory steps since treatment with kinase or phosphatase inhibitors blocks subsequent downstream signaling (Nürnberger et al., 1994; Jabs et al., 1997; Blume et al., 2000). Clearly, one of the initial targets of such inhibitors would be the receptor-like kinases and their associated kinases/phosphatases (Monaghan and Zipfel, 2012), which are involved in the MAMP/DAMP perception. Downstream of these receptor complexes are also intracellular phosphorylation events and cascades, such as calcium-dependent protein kinases (CDPKs) (Schulz et al., 2013) and mitogen-activated protein kinase (MAPK) cascades (Pitzschke et al., 2009; Andreasson and Ellis, 2010; Tena et al., 2011; Meng and Zhang, 2013).

MAPK cascades comprise a series of at least three kinases, with the MAPK kinase kinase (MAP3K) phosphorylating a MAPK kinase (MKK) that then subsequently phosphorylates specific MAPKs at conserved threonine and tyrosine motifs. This dual phosphorylation at so-called “TXY” motifs (i.e., threonine and tyrosine separated by typically a glutamic or aspartic acid) causes a conformational change that enhances the kinase activity. The MAPK itself then phosphorylates its specific substrates to alter gene expression or modulate the activity of enzymes/proteins. At least 4 MAPKs are known to be activated after MAMP perception in *Arabidopsis thaliana* (Bethke et al., 2012; Eschen-Lippold et al., 2012). So far, only a few targets of these MAP kinases are known and most are validated only through *in vitro* assays (Meng and Zhang, 2013). The MAPK, MPK6, phosphorylates Nitrate Reductase 2 (NR2/NIA2) (Wang et al., 2010), and 1-Aminocyclopropane-1-Carboxylate Synthase 6 (ACS6) (Liu and Zhang, 2004; Joo et al., 2008), leading to enhanced stability or activity of the enzyme and thereby to higher ethylene and NO levels, respectively. MPK4 and its substrate, MKS1, form a complex in unstressed tissues; upon phosphorylation, the MKS1 substrate is released from the complex and thought to effect transcription together with WRKY transcription factors such as WRKY33 (Andreasson et al., 2005; Qiu et al., 2008). WRKY33 is also targeted by two other MAPKs, MPK6, and MPK3, where phosphorylated WRKY33 appears to positively regulate expression of the *PAD3* (*Phytoalexin-Deficient 3*) gene. *PAD3* encodes a P450 enzyme (CYP71B15) that catalyzes the last step of the biosynthesis of camalexin, a major phytoalexin in Arabidopsis (Mao et al., 2011). Other MAPK substrates with roles in stress responses include the transcription factors ERF104 (Bethke et al., 2009), ERF6 (Meng et al., 2013) and VIP1 (Djamei et al., 2007), the universal stress proteins PHOS32/34 (Merkouropoulos et al., 2008), as well as the Tandem Zinc Finger protein 9 (TZF9) (Maldonado-Bonilla et al., 2014). However, the diversity of MAPK functions in multiple cellular pathways suggests that many more MAPK substrates remain to be discovered.

Here, we studied transgenic gain-of-function *Arabidopsis thaliana* plants expressing a *Petroselinum crispum* constitutively active MKK5 protein under the control of a dexamethasone (DEX)-inducible promotor. Kinase assays following immunoprecipitation with specific MAPK antibodies have shown that transient expression in Arabidopsis of this heterologous MKK5 activated specifically two of the stress activated MAPKs, MPK3, and MPK6 but not MPK4/11 (Bethke et al., 2009). An “omics” experiment encompassing both metabolomics and proteomics was performed on the transgenic plants, which revealed a strong reprogramming of the defense metabolome and proteome. A novel phosphoproteomics procedure (Lassowskat et al., 2013) was used to enrich for phosphoproteins from leaf material, enabling us to uncover hundreds of putative phosphoproteins with altered abundance downstream of MPK3/6 activation. The presence of known MAPK substrates in this list of phosphoproteins supports the notion that novel MPK3/6 substrate candidates can be found using this strategy. Some of these candidates are regulators for the observed metabolomics reprogramming. Hence, by linking proteomics and metabolomics, this study reveals a whole range of adaptations *in planta* by just activating the two kinases, MPK3 and MPK6.

## Materials and methods

### Plant material and growth

*Arabidopsis thaliana* (Col-0) was transformed with a constitutively-active variant of MKK5 (designated as MKK5^DD^ or DD) from *Petroselinum crispum* (Lee et al., 2004), under the control of a dexamethasone(DEX)-inducible promoter (Aoyama and Chua, 1997). As a control, the Col-0 genotype was additionally transformed with a kinase-inactive MKK5 mutant (designated as MKK5^KR^ or KR). These two transgenic lines are hereafter abbreviated as Col-0 DD or Col-0 KR, respectively. For the MKK5^DD^ construct, transformation was also performed with the MAPK mutants, *mpk3* (Miles et al., 2005) and *mpk6-3* (Wang et al., 2008), the ethylene-insensitive mutants, *ethylene-insensitive2* (*ein2-1*) and *ethylene-insensitive3* (*ein3-1*)/*ein3-like 1* (*eil1-1*) double mutant (An et al., 2010), as well as the NADPH oxidase mutant (*respiratory burst oxidase homologD, rbohD*) (Torres et al., 2002). After selection for hygromycin resistance, putative transformants were validated for DEX-inducible expression of the myc-tagged MKK5 protein by western blot analysis. This resulted in the following independent transformants: 4 Col-0 KR, 14 Col-0 DD, 2 *mpk3* DD, 4 *ein2* DD and only one transgenic line for *mpk6* DD, *rbohD* DD or *ein3/eil1* DD. Stability of transgene expression was monitored by western blotting in the F2 generation and segregation of the DEX-inducible cell death phenotype (for the MKK5^DD^ transgenic lines) was tested in the F3 generation. After these initial characterizations, a representative line—with comparable DEX-inducible expression level of the MKK5 protein—was selected for each genotype for subsequent analysis.

Seeds were sown on soil, stratified for 2 days at 4°C, and after transfer to individual pots, the plants were eventually maintained under short-day conditions (8 h day light, 200 μE, 23°C) for 6 weeks prior to DEX treatment (20 μM DEX in 0.0075% SILWET L-77). Three biological replicates were collected with each sample (genotype or timepoint) consisting of pooled leaf material from six different plants.

### Western blot

Protein extraction and immunoblot with anti-*p*TE*p*Y (α-phospho-p44/42-ERK; CST, www.cellsignal.com) to detect activated forms of the MAPKs or c-myc epitope to detect the MKK5 proteins were performed as described (Ranf et al., 2011).

### Proteomics analysis

#### Protein extraction and phosphoprotein enrichment

Details to total protein and phosphoprotein enrichment via the Prefractionation-assisted Phosphoprotein Enrichment (PAPE) procedure can be found in Lassowskat et al. (2013). PAPE is a two-step fractionation with 40% ammonium sulfate precipitation that apparently enriches phosphoproteins (Lassowskat et al., 2013), followed by a metal oxide affinity chromatography (MOAC) step (Wolschin and Weckwerth, 2005). Typically, 25 g of frozen leaf material were ground to a fine powder in liquid nitrogen and extracted by vigorous shaking (20 min, 4°C) with three volumes (w/v) of extraction buffer (100 mM HEPES-KOH, pH 7.5, 5% glycerol, 5 mM EDTA, 0.1% (v/v) ß-mercaptoethanol, 1% (v/v) protease and phosphatase-inhibitor II and III mix from Sigma-Aldrich). After centrifugation (3220 × g, 4°C, 15 min) and filtering through a 0.45 micron Rotilabo® cellulose mixed ester (CME) filter (Roth, http://www.carlroth.com), the supernatant was either used for 40% ammonium sulfate precipitation (first step of PAPE as described; Lassowskat et al., 2013) or mixed with Tris-EDTA-buffered phenol (for the total protein fraction). The lower organic phase after centrifugation (3220 × g, 4°C, 15 min) was re-extracted (with an equal volume of 100 mM Tris-HCl, pH8.4, 20 mM KCl, 10 mM EDTA, 0.4% ß-mercaptoethanol). Proteins in the phenol phase were precipitated overnight (−20°C) with five volumes of cold precipitation solution (methanol containing 100 mM ammonium acetate). The protein pellet (3220 × g, 4°C, 15 min) was rinsed with precipitation solution and twice with a cold mixture (−20°C) of 80% (v/v) acetone/20% Tris-HCl, pH 7.5. After 5 min drying, the pellet was resuspended in shotgun buffer (50 mM Tris-HCl, pH 8.5, 8 M urea) to obtain the total protein extract.

For the second step in the PAPE procedure, strict adherence to the protein:metal matrix ratio is essential for the MOAC step. Typically, a 1.5 ml protein sample (0.5 mg/ml) was incubated with 40 mg of Al(OH_3_) (Sigma-Aldrich) that had been washed and equilibrated in MOAC incubation buffer. After 30 min (4°C) rotation, the metal oxide was precipitated (18514 × g, 2 min) and rinsed four times with incubation buffer. The proteins were eluted twice (800 and 400 μl) with tetrapotassium pyrophosphate (TKPP) buffer (8 M urea, 100 mM TKPP, pH 9.0) for 45 min at room temperature. The pooled eluates were centrifuged twice (18514 × g, 2 min, 15°C), to pellet any remaining matrix, and concentrated with centricon filter devices (3 kDa cut-off; Millipore, http://www.merckmillipore.com). Proteins were precipitated with a 2D-CleanUp kit (GE Healthcare, http://www.gelifesciences.com), according to the manufacturer's instructions, and solubilized in shotgun buffer.

#### In-solution digestion

Protein concentration was determined by 2-D Quant Kit (GE Healthcare, www.gelifesciences.com) and the proteins were reduced (100 mM DTT, 100 mM Tris-HCl, pH 7.8, for 1 h) and alkylated (200 mM iodoacetamide, 100 mM Tris-HCl, pH 7.8, for 1 h). The solution was diluted to an end concentration of 0.5 M urea with 50 mM NH_4_HCO_3_ (pH 8) and digested overnight with sequencing grade trypsin (Promega, www.promega.com) at a ratio of 1:50 at 37°C. Peptides (max. 200 μL) were desalted on C18 columns (for up to 30 μg peptide samples, PepClean™, Thermo Scientific, www.piercenet.com; or for up to 1 mg peptide samples, C18 Protea Spin Tips, Protea Biosciences, https://proteabio.com/) according to the supplier's instructions and reconstituted in 5% ACN, 0.1% TFA.

#### Mass spectrometry (proteomics)

Tryptic digests were analyzed with an LC-MS system consisting of a nano-LC (Easy-nLC II, Thermo Scientific, www.proxeon.com) coupled to a hybrid-FT-mass spectrometer (LTQ Orbitrap Velos Pro, Thermo Scientific, www.thermoscientific.com). Peptide separations were performed on a C18 column (EASY column; 10 cm, ID 75 μm, particle diameter 3 μm) at a flow rate of 300 nL/min and a linear gradient of 5–40% B in 150 min for total protein measurements and 300 min for measurement of the phosphoprotein-enriched fractions (A: 0.1% formic acid in water, B: 0.1% formic acid in ACN). A voltage of +1.9 kV was applied to electrospray peptide ions. A capillary temperature of 275°C for peptide transfer and a lock mass of 445.120024 m/z were used. Precursor mass scanning was performed from 400 to 1850 m/z in the Orbitrap with a resolution of 30,000 and the 20 most intense precursor ions were selected for subsequent CID fragmentation in the linear trap quadrupole mass analyzer (LTQ). Singly charged ions were rejected from fragmentation. Dynamic exclusion was enabled (Repeat Count: 1, Repeat Duration: 20 s, Exclusion List Size: 500, Exclusion Duration: 30 s).

#### Spectral data analysis

Each sample (genotypes/treatments (i.e., transgene)/timepoints) is based on three biological replicates, which are each measured twice (resulting in six values per sample). The resulting MS raw data were analyzed with the Progenesis LC-MS software (Nonlinear Dynamics Limited, www.nonlinear.com). After alignment and feature detection, normalization was applied automatically to all features as recommended by the Progenesis LC-MS software manual. Resulting features were filtered for an ANOVA *p*-value of <0.05 and fold-change of >2.0 (between the MKK5^DD^ and MKK5^KR^ plants). Available MS spectra with rank 10 and better were searched against an A. thaliana protein database based on TAIR10 (www.arabidopsis.org) using an in-house Mascot server (with the following parameters: precursor mass tolerance: 7 ppm, fragment mass tolerance: 0.8 Da, missed cleavages: 2). Carbamidomethylation of cysteine was set as a static modification. Variable modifications were oxidation (M), acetylation of protein N-terminus, deamidation (NQ), and phosphorylation (STY). Peptides with Mascot scores less than 20 were rejected. Protein grouping and quantitation from non-conflicting peptides was enabled. Normalized abundance relative to maximum (Tables S04–S09) was calculated based on normalized abundance from Progenesis LC-MS. Maximum value of each protein (from 0 to 24 h samples) was set as one, with all other values being relative to this. Phosphopeptides were identified with the Proteome Discoverer 1.4 software, which includes the phosphoRS 2.0 algorithm (Thermo Fisher Scientific) for phospho-site mapping. A false discovery rate (FDR) was calculated by searching a “decoy” database containing all the target database sequences in reverse order. Peptide-spectrum match (PSM) was set at a *q* < 0.05 (i.e., a corrected significance threshold employing the Benjamini-Hochberg FDR procedure to control for a family-wise error rate).

#### Targeted proteomics analysis

The LC method with 300 min gradient as well as the MS acquisition method was identical to point Mass spectrometry (proteomics) with the following changes. A global parent mass list was used (Table S20) and dynamic exclusion was disabled. Parent masses were extracted from previous measurements of phosphopeptides. Spectral data analysis was performed with Proteome Discoverer 1.4 according to parameters described in point Spectral data analysis. The number of PSMs was calculated as a sum from measurements of the four samples (collected 4, 5, 7, 8 h after DEX treatment).

### Metabolomics analysis

#### Metabolite extraction

Frozen leaf material was homogenized in liquid nitrogen and weighed (50 ± 2 mg) into a pre-cooled 2-ml polypropylene tube. After addition of 80% methanol (200 μL), pre-cooled at −80°C and spiked with indole-3-acetyl-L-valine (1 μM), o-anisic acid (1 μM), and kinetin (2 μM) as internal standards the sample was allowed to reach room temperature under vigorous shaking within 5 min. The resulting mixture was sonicated (10 min, 20°C) and centrifuged at 11,200 ×g (10 min, 16°C). The sediment was re-extracted with 80% methanol (200 μL). Both supernatants were combined and evaporated to dryness under reduced pressure at 40°C using a vacuum centrifuge. The remaining residue was thoroughly reconstituted in 30% methanol (150 μL), sonicated (15 min, 20°C) and centrifuged at 11,200 × g (10 min, 16°C). An 80-μl aliquot of the supernatant was transferred into an autosampler vial and subjected to LC/MS analysis in both positive and negative ion mode. For quantification of abundant metabolites a 30-μl aliquot of the supernatant was diluted with 120 μl methanol/water, 3/7 (v/v) and subjected to LC-MS analysis in negative ion mode.

#### Mass spectrometry (metabolomics)

Leaf extracts were analyzed with a LC-MS system consisting of an UPLC (Acquity, Waters, www.waters.com) coupled to a hybrid quadrupole time-of-flight mass spectrometer (micrOTOF-Q I, Bruker Daltonics, www.bruker.com) equipped with an Apollo II electrospray ion source. Chromatographic separations were performed at 40°C on a HSS T3 column (100 × 1.0 mm, particle size 1.8 μm, Waters) applying the following binary gradient at a flow rate of 150 μL min^−1^: 0–1 min, isocratic 95% A (0.1% formic acid in water), 5% B (0.1% formic acid in acetonitrile); 1–10 min, linear from 5 to 60% B; 10–12 min, isocratic 95% B; 12–14 min, isocratic 5% B. The injection volume was 2.6 μL (full loop injection). Eluted compounds were detected from *m/z* 100–1000 in positive (negative) ion mode using the following instrument settings: nebulizer gas, nitrogen, 1.6 bar; dry gas, nitrogen, 6 l/min, 190°C; capillary, −5000 V (+4000 V); end plate offset, −500 V; funnel 1 RF, 200 Vpp; funnel 2 RF, 200 Vpp; in-source CID energy, 0 V; hexapole RF, 100 Vpp; quadrupole ion energy, 3 eV; collision gas, argon; collision energy, 3 eV (10 eV); collision RF 200/400 Vpp (timing 50/50); transfer time, 70 μs; pre pulse storage, 5 μs; pulser frequency, 10 kHz; spectra rate, 3 Hz. Mass spectra were acquired in centroid mode. Calibration of the *m/z* scale was performed for individual raw data files on lithium formate cluster ions obtained by automatic infusion of 20 μl 10 mM lithium hydroxide in isopropanol/water/formic acid, 49.9/49.9/0.2 (v/v/v) at a gradient time of 12 min using a diverter valve. For aquisition of collision-induced dissociation mass spectra appropriate precursor ions were selected using an isolation width of ± 4 *m/z* and fragmented inside the collision cell applying collision energies in the range of 10–40 eV. Product ions were detected using the same parameter settings as described above, except for spectra rate (1.5 Hz) and collision RF [100/200 Vpp (timing 50/50)].

#### Non-targeted data analysis and metabolite annotation

A total of 48 undiluted methanolic leaf extracts [genotype × treatment time × replicate = (Col-0 DD, Col-0 KR) × (0, 4, 8, 12, 16, 20, 24, 36 h) × 3] were analyzed by UPLC/ESI-QTOFMS in positive and negative ion mode resulting in a total of 96 raw data files. To judge analytical quality and estimate alignment parameters retention times and responses of spiked internal standards were determined in prior to non-targeted data analysis (retention time deviation ≈ ±2.5 s, coefficient of variation of internal standard responses ≤11%). Raw data files were converted into mzData format using CompassXPort (Bruker Daltonics) and processed for each ion mode in two batches of 48 raw data files using the R package XCMS (Smith et al., 2006). Raw data files were arranged in 16 sample classes according to genotype and treatment time. Feature detection was performed using the centWave algorithm [sntresh = 3, prefilter = (3, 100), ppm = 25, peak width = (5, 12)]. Alignment was accomplished by consecutively applying the XCMS functions *group.density* (minfrac = 1, bw = 2 and mzwid = 0.05), *retcor* (span = 1, missing = 0, extra = 0), and *group.density* (minfrac = 1, bw = 1 and mzwid = 0.03). Missing feature intensities were filled in using the XCMS function *fillPeaks*. The resulting intensity matrix (feature × sample) was log2-transformed and subjected to Two-Way ANOVA. Features with a significant genotype-treatment time interaction (*p* < 0.01) were filtered by intensity (maximum intensity ≥ 2^14^ counts). The resulting feature sets (positive and negative ion mode) were used for reconstruction and annotation of the underlying compound mass spectra which was aided by the R package CAMERA (Kuhl et al., 2012) and manual analyses using DataAnalysis 4.0 (Bruker Daltonics). High-resolution CID mass spectra of annotated quasi-molecular ions were acquired. Elemental compositions were calculated on accurate *m/z* of quasi-molecular ions within an error range of 10 ppm and filtered by relative isotope abundance, number of double bond equivalents and electron parity using the SmartFormula algorithm implemented in DataAnalysis 4.0. Putative elemental compositions were further checked for consistency by analysis of elemental compositions of fragment ions. Compounds were then putatively annotated by interpretation of characteristic fragment ions and neutral losses. Annotation of a total of 23 metabolites could be verified by analysis of reference compounds. Analytical data and mass spectral characterization is given in Tables S1, S2. Finally, all annotated compounds were manually quantified by integration of extracted ion chromatograms using QuantAnalysis 2.0 (Bruker Daltonics) and retention times/quantifier ions from Table S19.

## Results

### Transgenic expression of a constitutively active MKK5 (MKK5^DD^) leads to activation of MPK3 and MPK6

**Figure 1:**
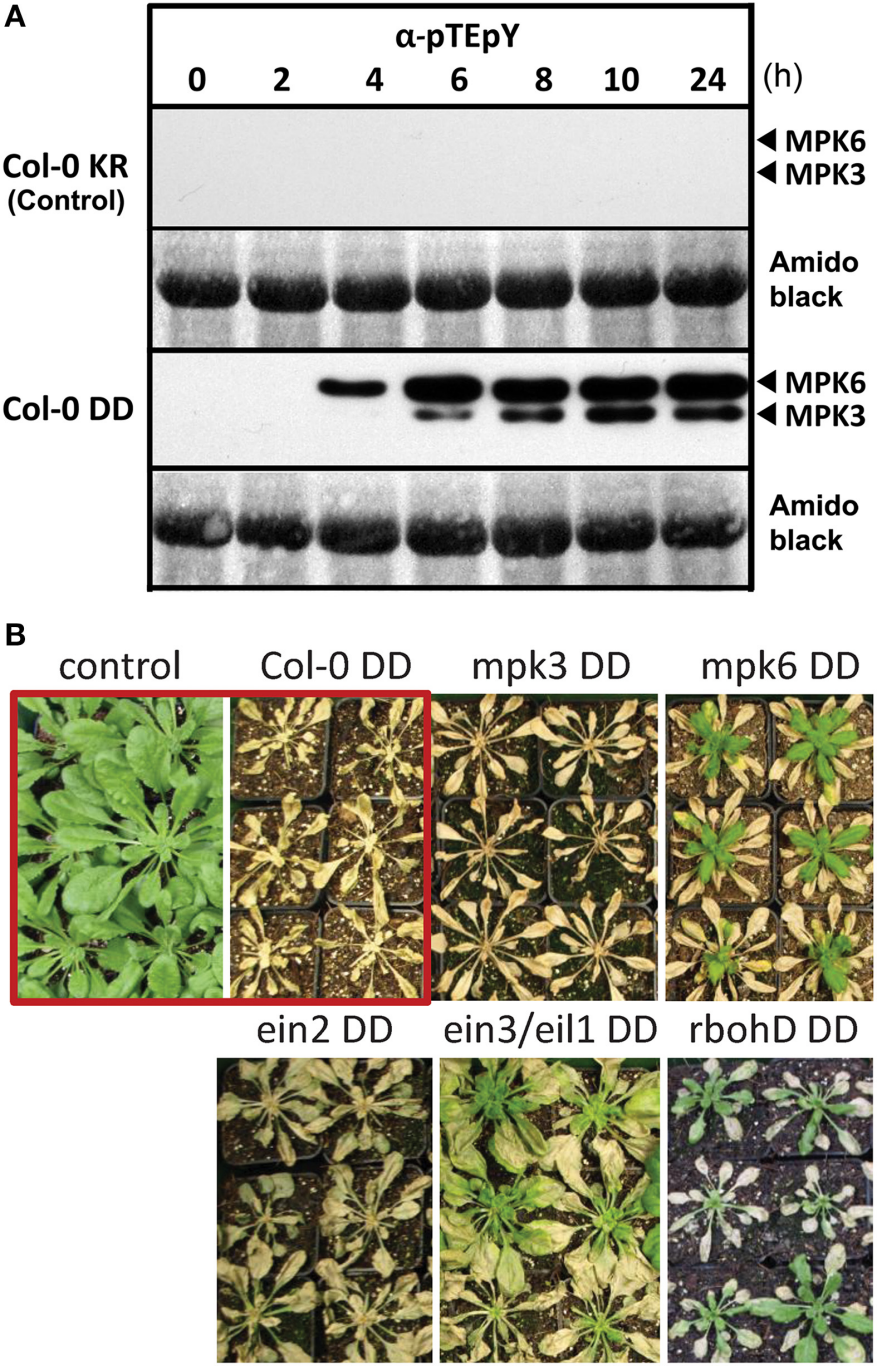
**Activation of the MAP kinases, MPK 3 and MPK6, by DEX-inducible expression of a constitutively active MAPK kinase, MKK5**. **(A)** Six week old plants were sprayed with 20 μM DEX to induce expression of a constitutively active MAPK kinase 5, MKK5^DD^, in Col-0 ecotype background (abbreviated as Col-0 DD). Leaves were harvested at the indicated time points after DEX treatment and used for immunodetection of phosphorylated MAPKs (α-*p*TE*p*Y). The expected positions of MPK3/6 are marked. Col-0 KR designates the corresponding control plants expressing a kinase-inactive version of MKK5. **(B)** Prolonged MPK3/6 activation, after DEX treatment, lead to death of the plants—visible as tissue collapse after 24 h. (Note that for some lines, new leaves emerged from the central meristem some days after the DEX treatment). Photos of the plants were taken 2 week after DEX treatment. The core experiment (within red box) compares the Col-0 KR (control) with Col-0 DD (active MKK5^DD^) lines. Additional transgenic lines expressing the MKK5^DD^ in the indicated mutant background are also included (For detection of phosphorylated MAPKs for these lines, please see Figure S1).

Substitution of two serines by “phosphomimicking” aspartic acid (D) in the kinase activation loop of the parsley MAPK kinase 5 (MKK5) generates a constitutively active kinase (abbreviated as MKK5^DD^) that can phosphorylate and activate downstream MAPKs (Lee et al., 2004). Transgenic *Arabidopsis thaliana* (Col-0) plants were created with a dexamethasone (DEX)-inducible expression of this heterologous MKK5^DD^ (hereafter named Col-0 DD) (see material and methods for generation and validation of the transgenic plants). Two immunoreactive bands representing phosphorylated MAPKs are detected in plant extracts of such transgenic plants after 4–6 h of DEX treatment (Figure 1A), which is not seen in corresponding control plants (Col-0 KR) that express the kinase-inactive version of MKK5 (MKK5^KR^). Previous experiments using MAPK-specific antibodies have defined these two bands to be MPK3 and MPK6 (Bethke et al., 2009) and this is now further confirmed by the loss of one of the MAPK bands in the respective *mpk3* or *mpk6* mutant background (see Figure S1).

A prolonged activation of MPK3 and/or MPK6 leads to tissue collapse within 24–48 h, and to complete death after 4–7 days post DEX treatment (Figure 1B). Similar experimental set-up with the tobacco MKK5 ortholog has pointed to subsequent downstream ethylene and reactive oxygen species (ROS) production (Ren et al., 2002; Liu and Zhang, 2004). To evaluate the influence of such downstream signaling molecules and also the individual contribution of MPK3 or MPK6, the MKK5^DD^ construct was also transformed in *mpk3, mpk6, respiratory burst oxidase homologD* (*rbohD*), *ethylene-insensitive2* (*ein2*) mutants, as well as the *ethylene-insensitive3/ein3-like1* (*ein3/eil1*) double mutant. DEX-induced death of the plants was seen in all these genetic backgrounds. However, new leaves developed from the central meristem for the *mpk6, rbohD*, and *ein3/eil1* double mutants 7–14 days later (Figure 1B); thus pointing to roles of the corresponding genes in the cell death phenotype. Taken together, the transgenic system described here simulates MPK3/6 activation after pathogen attack without any direct exposure to pathogens or any pathogen-derived molecules (e.g., MAMPs).

### Metabolic outcome of simulated pathogen defense

**Figure 2:**
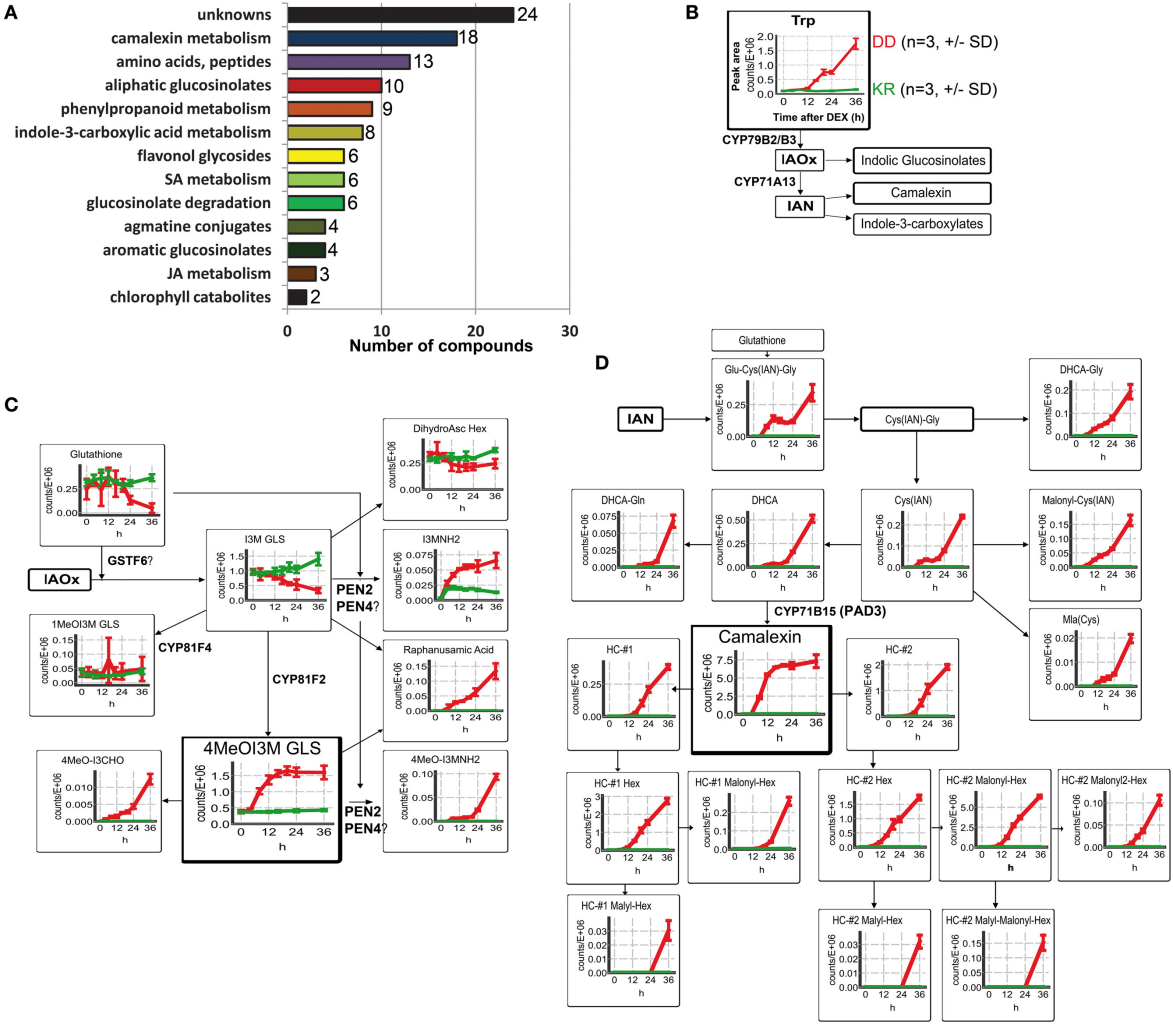
**Modulation of metabolic defense response upon phosphorylation of MPK3/6**. Wild type Col-0 DD and control plants were harvested 0–36 h after DEX treatment and methanolic extracts from leaves were analyzed by UPLC/ESI-QTOF-MS in both positive and negative mode. **(A)** Classification of the annotated metabolites. **(B)** General scheme of the biosynthesis of tryptophan (Trp)-derived metabolites. Change in levels of the precursor, Trp, is shown as mean of peak area ± standard deviation (SD) of three biological replicates. Accumulation kinetics for the control plants (Col-0 KR) are in green and for Col-0 DD in red. (IAOx = indole-3-acetaldoxime; IAN = indole-3-acetonitrile). **(C)** Changes in the levels of indolic glucosinolate (GLS) metabolites derived from IAOx are depicted as described in **(B)**. **(D)** Changes in the levels of metabolites derived from IAN, i.e., camalexin derivatives and precursors. (HC, Hydroxycamalexin; DHCA, Dihydrocamalexic acid. For other abbreviations, see Table S1). Note that for all the graphs in **(B–D)**, the y-axis represents the relative abundance of the metabolites (peak area of the quantifier ion, Table S19) and the x-axis the time (hours, h) after DEX treatment.

To investigate the impact of artificial MPK3/6 activation on plant defense metabolism without any complications from pathogens, we performed non-targeted metabolite profiling for semi-polar secondary metabolites. Samples were collected 0, 4, 8, 12, 16, 20, 24 and 36 h after DEX treatment in three independent experiments. These timepoints were selected to cover the initial time period where the MPK3/6 activation are just detectable (~4–6 h) (Figure 1A) until (and after) the initiation of tissue collapse (24–48 h). Note that at 36 h, most tissues from the MKK5^DD^ plants are partially collapsed and flaccid, but not completely dead (i.e., brown and dry, see Figure S1B). A total of 12,913 and 10,731 molecular features were extracted from LC-MS data acquired in the positive and negative ion mode, respectively. After setting an intensity filter cutoff (>2^14^ counts) and Two-Way ANOVA (*p* < 0.01), 853 and 724 differential features remained, which were further subjected to metabolite annotation (for analytical data see Tables S1, S2). In combination with known leaf metabolites this resulted in a total of 113 compounds, including 24 unidentified substances (Figure 2A). Many known defense-related compounds accumulated to high levels (see Figure 2A for compound classification and Table S1 for a complete list). The most prominent and numerous are a variety of tryptophan (Trp)-derived defense metabolites (camalexin and indole glucosinolate derivatives) as well as Trp-derived indole-3-carboxylic acid derivatives (Figure 2B and Figure S2A).

Various indole glucosinolate (GLS) derivatives, initially described to be associated with herbivore resistance (Barth and Jander, 2006; Winde and Wittstock, 2011; Stauber et al., 2012; Schweizer et al., 2013) and subsequently to penetration resistance by filamentous pathogens (Bednarek et al., 2009; Clay et al., 2009), were found to accumulate rapidly. In particular, accumulation of indole-3-ylmethylamine (I3MNH_2_) and its 4-methoxy derivative (4MeOI3MNH_2_) were observed already after 4–6 h DEX treatment (Figure 2C). This is almost concomitant with the MPK3/6 activation (cf. Figure 1A). Similarly, levels of raphanusamic acid and 4MeOI3M GLS also start to rise within 6 h. By contrast, the precursor metabolite, indole-3-ylmethyl (I3M) GLS, is reduced ~8 h after DEX treatment, possibly as a consequence of heightened flux into the downstream metabolites. I3M GLS is synthesized from indole-3-acetaldoxime (IAOx), which can additionally be converted into indole-3-acetonitrile (IAN) by the cytochrome P450 enzyme CYP71A13 (Nafisi et al., 2007) (Figure 2B). IAN can further be conjugated to glutathione, and converted into the phytoalexin, camalexin. Indeed, camalexin, and its precursors rapidly accumulated 6 h after DEX treatment, with camalexin abundance reaching a plateau phase within 12 h, which is followed by an increase of various hydroxy-camalexins (HC) and their conjugates (~12–24 h) (Figure 2D). Since these compounds can already be detected prior to any obvious macroscopic tissue damage, their accumulation is unlikely to be caused by tissue wounding or cell death. Hence, artificial activation of MPK3/6 is sufficient to cause massive accumulation of the major Arabidopsis antimicrobial metabolites.

Induced metabolite accumulation was also seen for some anabolites and catabolites of Met-derived GLS, as well as many derivatives of indole-3-carboxylic acid, agmatine conjugates and various aromatic amino acids. By contrast, little to no changes could be seen for flavonol glycosides and hydroxycinnamate esters (Figures S2A–F). However, some of the hydroxycinnamic acids were probably channeled into the accumulating feruloyl or coumaroyl agmatines (cf. Figure S2C). In addition to the previously described increase of ethylene production after MPK3/6 activation (Liu and Zhang, 2004), precursors and conjugates of defense-related phytohormones such as salicylic acid (SA) and jasmonic acid (JA) were also detected (Figures S2G,H). In agreement with the observed tissue collapse and cell death (Figure 1A), two non-fluorescent chlorophyll catabolites, NCC1 and NCC4, start to appear 12–14 h and are detected at high levels 36 h after DEX treatment (Figure S2I).

**Figure 3:**
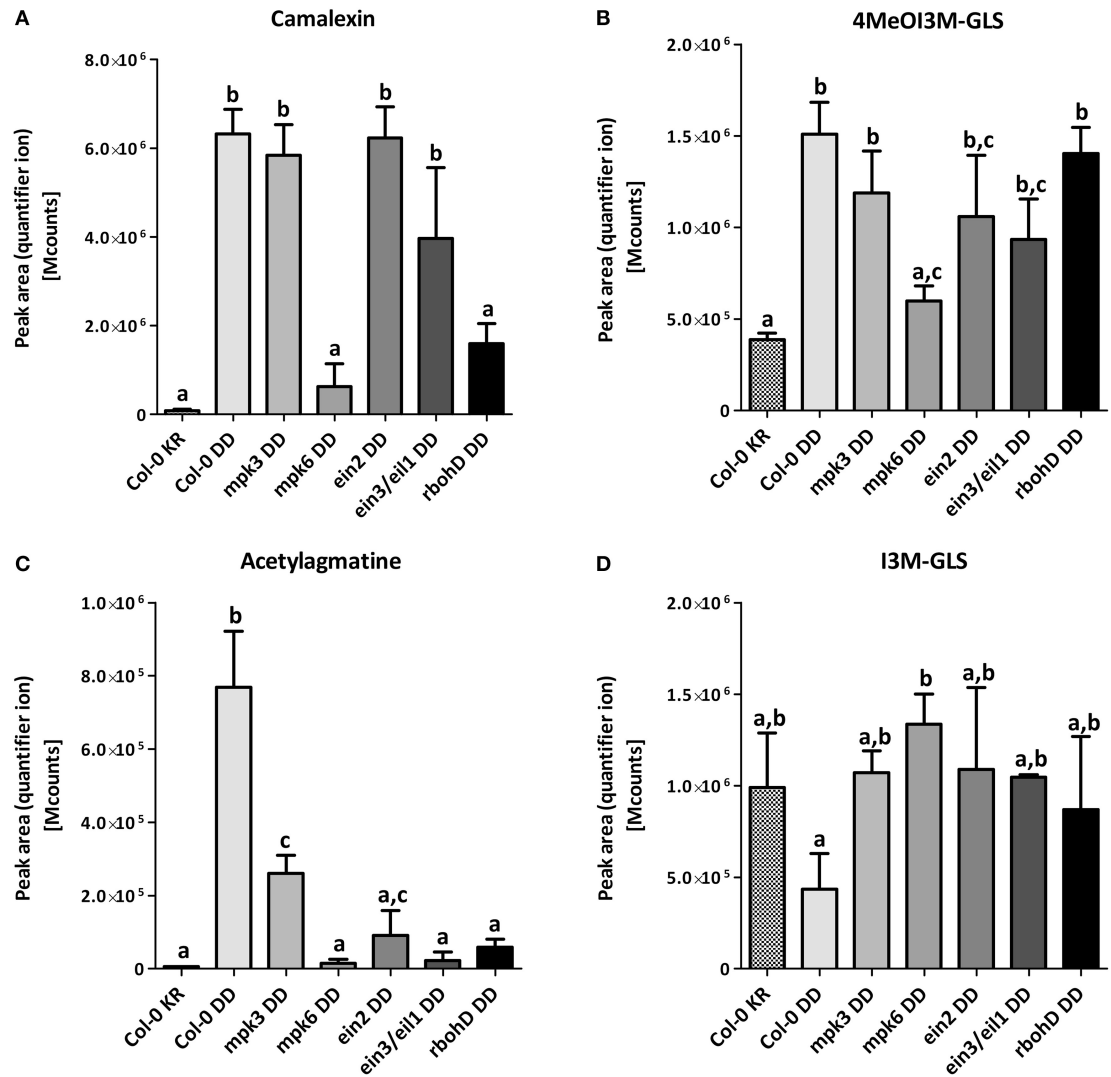
**Mutant background influences metabolome modulation**. Samples were taken 24 h after DEX treatment in three independent experiments. The peak areas of the quantifier ion (Table S19) for camalexin **(A)**, 4MeOl3M-GLS **(B)**, acetylagmatine **(C)** and I3M-GLS **(D)** were determined for relative quantification. Significance of changes was calculated by One-Way ANOVA with Tukey's multiple comparison post-test, where the different alphabets denote statistically distinct groups. Note that in B, the levels of 4MeOI3M-GLS in the *mpk6, ein2* or *ein3*/*eil1* mutants are not distinguishable and therefore additionally marked with the letter c.

To check which of the two MAPKs or whether downstream signaling through ethylene or ROS contributes to the accumulation of these metabolites, we compared in various mutant backgrounds the levels of three selected defense-related metabolites known to have antimicrobial activities against pathogens/microbes 24 h after DEX treatment. MPK6 is clearly required for the DEX-induced accumulation of camalexin, 4MeOI3M GLS and acetylagmatine (Figures 3A–C). On the other hand, MPK3—while not absolutely essential—contributes to reaching maximum acetylagmatine levels but appears to be dispensable for camalexin and 4MeOI3M GLS accumulation. Camalexin production is independent of ethylene signaling but required ROS generated by the NADPH oxidase, RBOHD (Figure 3A). By contrast, acetylagmatine accumulation required both ethylene and ROS signaling (Figure 3C); while 4MeOI3M GLS appears to require neither ethylene nor ROS from RBOHD (Figure 2B). On the other hand, no statistically significant changes between the tested mutants was observed (Figure 3D) for the 4MeOI3M GLS precursor, I3M GLS, which did not show strong DEX-induced accumulation (cf. Figure 2C). In summary, the DEX-induced accumulation of three major antimicrobial metabolites requires MPK6 activation but has differential requirements for downstream ethylene and ROS signaling to achieve similar metabolite levels as the wild-type plants.

The findings above indicate that MPK3/6 activation, without any direct exposure to pathogens or pathogen-derived MAMPs, is sufficient to trigger the accumulation of multiple antimicrobial and stress-related metabolites. This metabolic reprogramming must be regulated through the actions of MPK3/6 on their substrates as well as a change in the overall proteome. It therefore pinpoints the importance of MAPKs in activating defense reactions to counteract invading pathogens or pests.

### Changes in the total proteome following MPK3 and MPK6 activation

**Figure 4:**
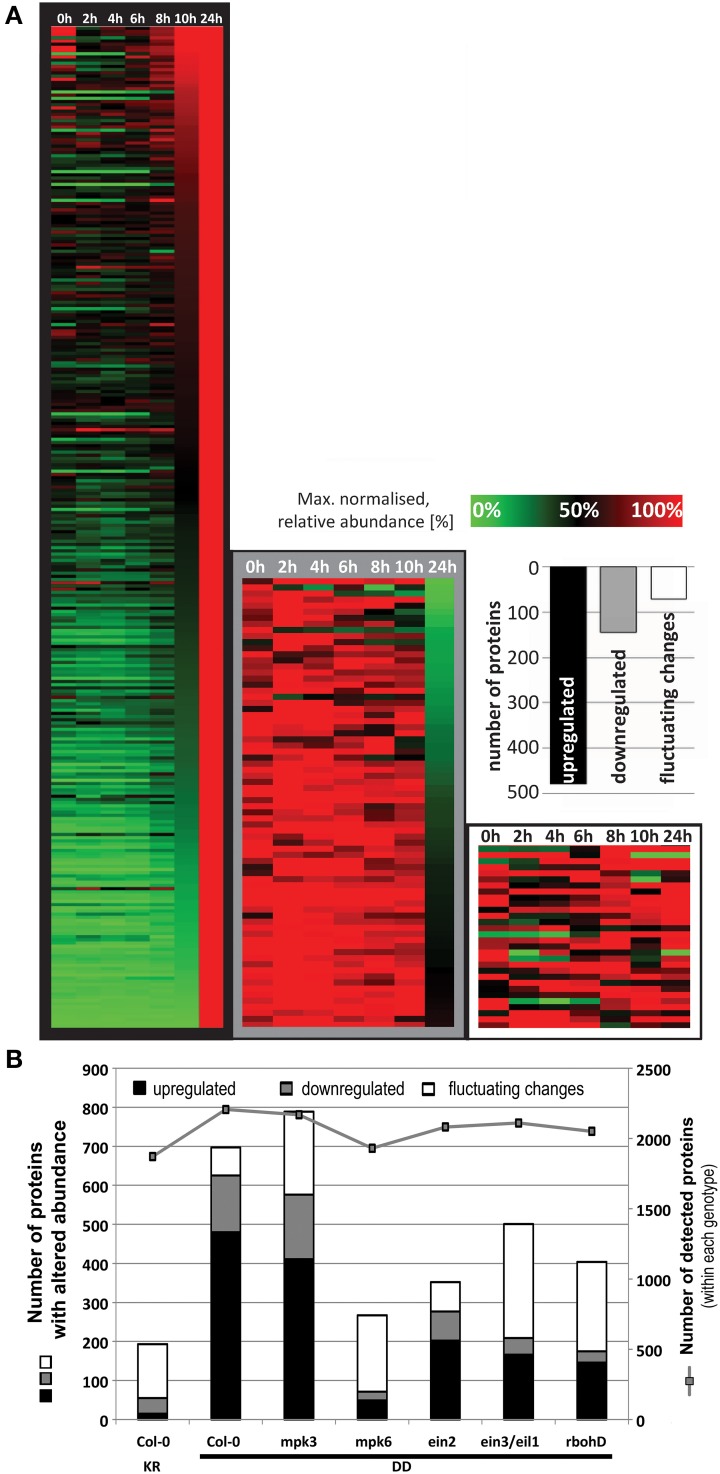
**Regulation of global proteome after DEX treatment to induce MPK3/6 activation. (A)** The relative abundance was normalized to the maximum value for each protein and a heat map generated by hierarchical clustering according to the MeV (Multiexperiment Viewer) algorithm. This resulted in a cluster of 480 up-regulated proteins, another with 145 down-regulated proteins and a third cluster of 72 proteins showing fluctuating changes. **(B)** Comparison of the total number of proteins with altered abundance between genotypes. To exclude any bias when comparing genotypes, total numbers of proteins detected within each genotype (right y-axis), which is relatively constant (~2000 throughout all genotypes), are also shown.

To elucidate the defense metabolism reprogramming by MPK3/6 activation, we performed a global proteome analysis of leaf material collected at 0, 2, 4, 6, 8, 10, 12, and 24 h after DEX spraying of the transgenic MKK5^DD^ plants compared to the MKK5^KR^ control plants. The time points were selected to cover the period where the rise in activated MAPKs (~4 h, Figure S1) and the rise in metabolites levels (typically starting at 6–12 h after DEX, *c.f*. Figure 2 and Figure S2) were detectable. Due to low protein yield, the 36 h timepoint was excluded. Triplicate samples (with each sample consisting of three plants pooled together) were collected for each genotype and time point. Two micrograms of extracted protein were digested with trypsin and separated with a 150-min LC gradient for LC-MS measurements. Quantitation of protein changes was performed with the Progenesis LC-MS software package and compared within each genotype. The heat map in Figure 4A illustrates the ~700 proteins with altered abundance (>2-fold, ANOVA, *p* < 0.05, for at least one of the measured time points) for the Col-0 genotype. Abundances for 480 and 145 proteins were gradually up- or down-regulated, respectively, during the 24 h period, while another group of 72 proteins showed fluctuating up- and down-regulation in an irregular manner (Figure 4A, Table S4). By contrast, the Col-0 KR plants had considerably less number of proteins with altered abundance (Table S3). With exception of the *mpk3* mutant with its list of 789 proteins, all other tested mutants had fewer proteins with altered abundance (i.e., 267–501, Figure 4B, Tables S5–S9). Since the total number of detected proteins is comparable between genotypes (i.e., ~2000 in all cases, Figure 4B), the reduced number of proteins showing altered abundance in some mutants is not due to overall lower protein detection in a particular sample. Rather, it suggests that the changes in protein abundance are dependent on ethylene and ROS signaling. For *mpk6*, the reduced number of protein changes suggests an important contribution of MPK6 but it may also be in part through diminished ethylene biosynthesis since MPK6 is a major MAPK for stabilizing ACC synthase levels through phosphorylation (Liu and Zhang, 2004; Joo et al., 2008).

**Figure 5:**
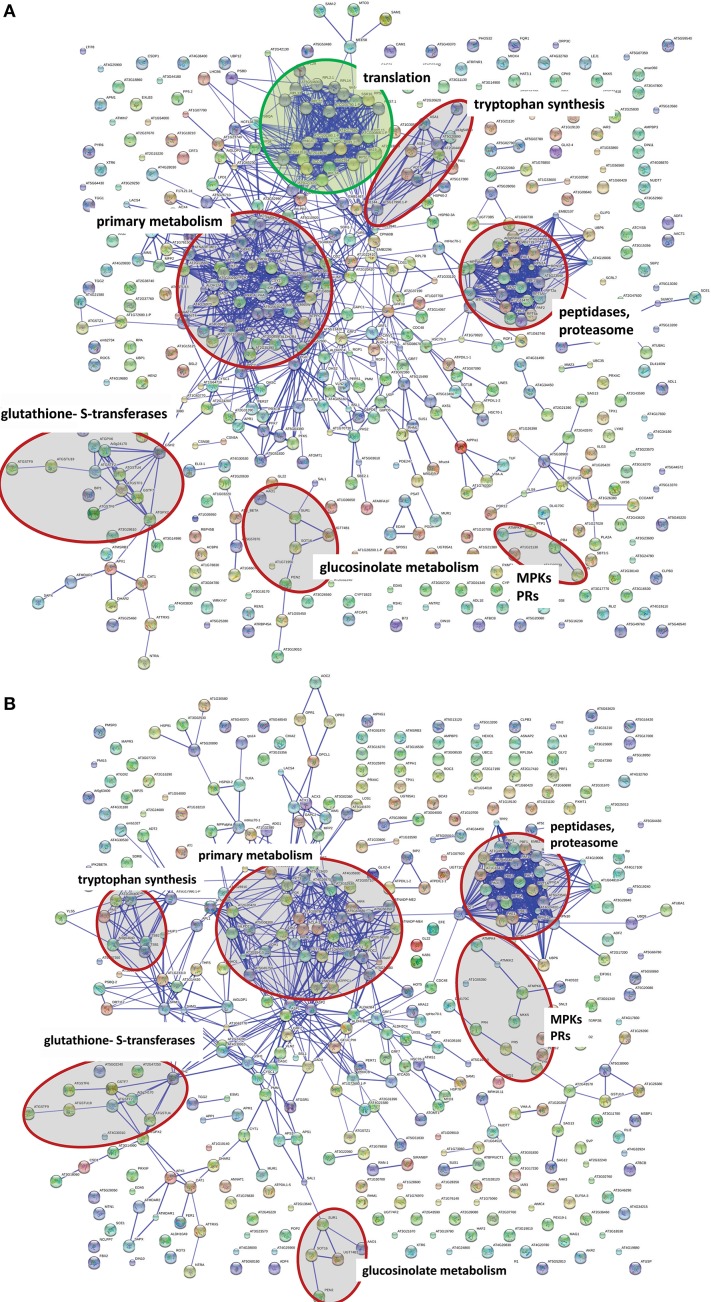
**Network comparison of protein extract between wild type DD and *mpk3***. DEX treatment causes a wide range of protein changes that can be organized in networks. Network calculation was done with STRING 9.05 (high confidence score 0.7, confidence view). Either the Arabidopsis locus identifier or the protein name abbreviation (where known) are shown. Selected functional clusters are highlighted in red. STRING network of the up-regulated protein in Col-0 DD plants **(A)** and the *mpk3* DD plants **(B)**. The green circle marks a cluster of proteins involved in protein translation, which is completely missing in the *mpk3* mutant.

Even though MPK3 and MPK6 are often mooted to have overlapping redundant functions, the results above point to a stronger role of MPK6 in mediating the observed global proteome changes. However, it is possible that the heterologous MKK5 used here may have some preference for MPK6 over MPK3 *in vivo*. Although *in vitro* kinase assays showed that recombinant parsley MKK5^DD^ can phosphorylate the Arabidopsis MPK3 and MPK6 equally well (not shown), the MPK3 phosphorylation by MKK5^DD^ in the *mpk6* background is indeed weaker and transient (Figure S1). Hence, the profile based on the number of proteins up- or down-regulated is, as expected, very similar between Col-0 and the *mpk3* mutant (*c.f*. Tables S4, S5). A “STRINGS interactome network” of the up-regulated proteins, which functionally clusters known and predicted protein-protein interactions (Franceschini et al., 2013), also revealed mostly similarities between Col-0 and *mpk3* (Figure 5). In agreement to the observed metabolome changes (Figure 2), up-regulated proteins with functions in primary metabolism, Trp biosynthesis, and glucosinolate metabolism, as well as gluthathione-*S*-transferases are prominently clustered. In addition, MAPK components, pathogenesis-related proteins and components of peptidase/proteasome degradation pathways are also pronounced. However, a cluster consisting of proteins involved in protein translation is completely missing in the *mpk3* mutant (Figure 5); amongst these are a number of ribosomal proteins (highlighted in yellow in Table S4). Thus, despite the apparent weaker MPK3 activation (as compared to MPK6) in the transgenic system, this unforeseen difference implies that MPK3 activation has a regulatory function on elements of the protein translational machinery, which is not replaceable by MPK6. Hence, besides their overlapping functions, MPK3 and MPK6 have distinct cellular targets.

### Identification of putative *in vivo* MAPK substrates

**Figure 6:**
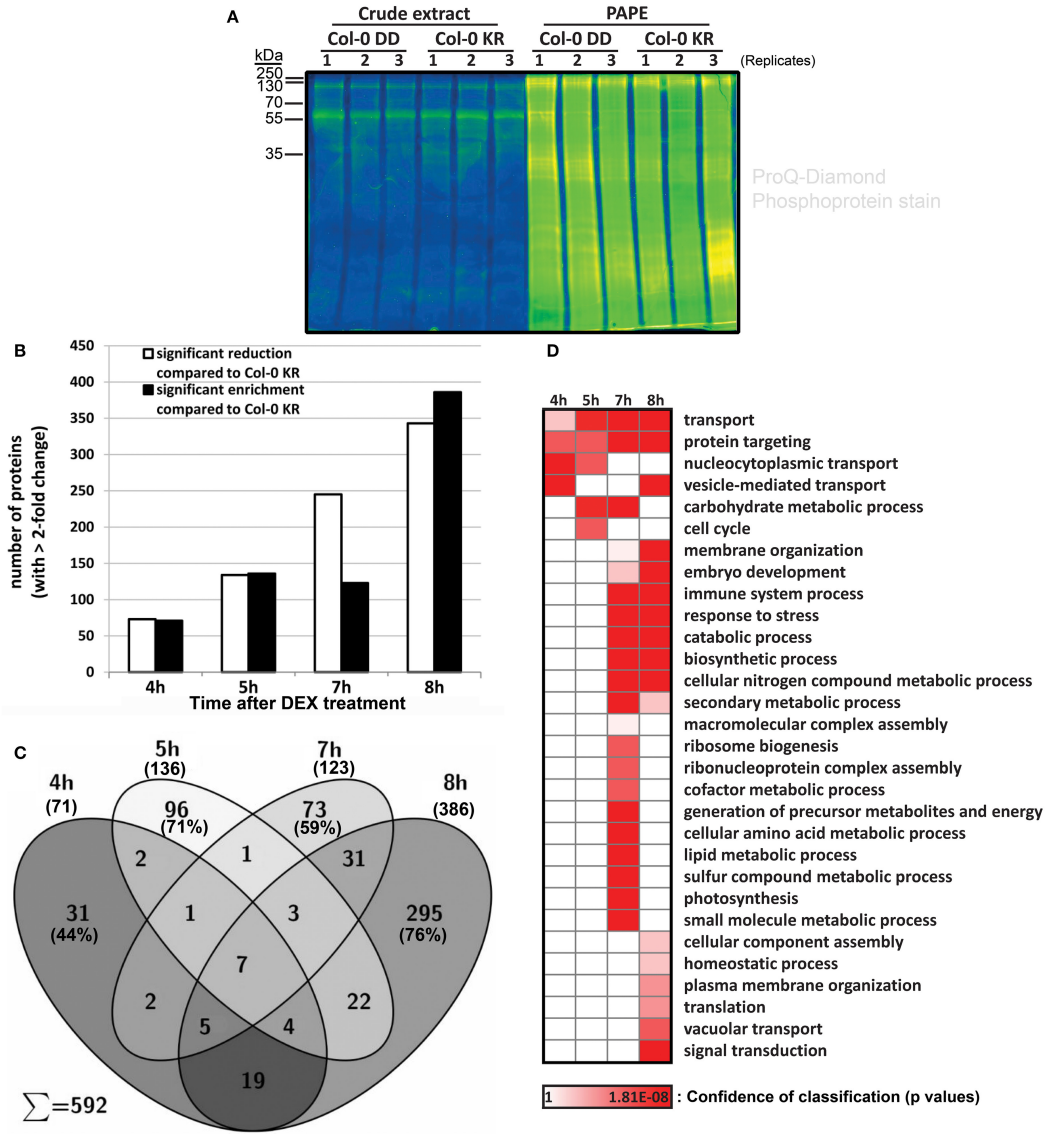
**Isolation and identification of phosphoproteins with the prefractionation-assisted phosphoprotein enrichment (PAPE) method**. Six week old plants of Col-0 DD and control (KR) were treated with DEX, harvested at different time points (4, 5, 7, 8 h) and subjected to PAPE procedure to isolate phosphoproteins. Analysis of these samples was performed with mass spectrometry and subsequent evaluation with Progenesis LC-MS. **(A)** Pro-Q Diamond phosphoprotein staining of ~10 μg of protein extracts before and after the PAPE procedure. The samples from the 5 h treatment were used exemplarily to illustrate the efficiency of the phosphoprotein enrichment through PAPE. **(B)** Number of proteins that showed a significant enrichment (black bars) or reduction (white bars) during PAPE compared to control plants (ANOVA *p* < 0.05 and maximum fold change of >2.0). **(C)** Venn diagram showing distribution of proteins with increased abundance (i.e., corresponding to black bars in **B**) during different time points after DEX treatment. **(D)** Enrichment of Gene Ontology (GO) biological process classifications in PAPE-protein extract for the putative phosphoproteins with increased abundance over the four time-points after DEX treatment. Annotation and calculation was done with STRING 9.05 (*p*-value with FDR correction <0.1, slim display). The confidence for the enrichment of the GO groups is indicated by the heat map of its *p*-value.

The findings above show that a simulated MPK3/6 activation reprograms the overall proteome and (defense) metabolome. From the rapid timing of the metabolic changes (Figure 2), one may postulate that a direct action of the activated MAPKs on their substrates is likely to be responsible for most of the observed effects. Thus, to identify MPK3/MPK6 substrates, we performed phosphoproteomics analysis using a novel “Prefractionation-Assisted Phosphoprotein Enrichment” (PAPE) procedure (Lassowskat et al., 2013). The PAPE method mitigates some of the difficulties of detecting phosphoproteins, which are typically of low-abundance, in photosynthesizing green plant tissues. Triplicate samples of pooled plant material were collected for the PAPE fractionation, and as illustrated by ProQ diamond phosphoprotein staining (Figure 6A), the PAPE procedure efficiently enriched for phosphoproteins. Four micrograms of the phosphoprotein-enriched proteins were trypsin-digested and each sample measured twice after a 300-min gradient separation by liquid chromatography. To distinguish between early and late (possibly “indirect”) MPK3/6 phospho-targets, we collected leaves at 4, 5, 7, and 8 h after DEX treatment (which overlaps with the initial and sustained MAPK activation profile, *c.f*. Figure 1).

More than 2000 proteins were detected for each fraction (*viz*. 4 h: 2733, 5 h: 2264, 7 h: 2621, 8 h: 2596; see Tables S10–S13). Comparison was carried out with Progenesis LC-MS label-free quantitation based on the peak area of the MS1 spectrum and filtered for significant changes in protein abundance (>2-fold, *p* < 0.05) between the MKK5^DD^- and the MKK5^KR^-expressing plants in the Col-0 background (Col-0 DD vs. Col-0 KR). While phosphoprotein enrichment is expected to increase detection of the putative MAPK substrates, several MAPK substrates are de-stabilized upon phosphorylation (Meng and Zhang, 2013) and may therefore exhibit decreased protein levels after being phosphorylated by MAPKs. Thus, putative phosphoproteins that are either up-regulated or reduced in levels when comparing the Col-0 DD and Col-0 KR are considered. For the 4 h treatment, 144 proteins of altered abundance compared to the Col-0 KR line are detected; this progressively increased to more than 729 proteins at 8 h (Figure 6B, Tables S10–S13). Since the 4 h time point marks the onset of the MPK3/6 activation (Figure 1), one may expect the 144 proteins with altered abundances at 4 h (i.e., 73 down- and 71 up-regulated) to represent the initial MPK3/6 targets.

For simplicity, we will concentrate here only on the phospho-enriched proteins with increased abundance in the MKK5^DD^ plants. Besides the general increase in number of enriched proteins, the protein compositions also changed between the time points—with 44% (4 h), 71% (5 h), 59% (7 h), and 76% (8 h) of the putative phosphoproteins being unique to each time point (Figure 6C). Although not all the putative phosphoproteins detected are direct MAPK substrates and particularly those from the later time points are likely “indirect” phosphoproteins, the above observation suggests a progressive change in the phosphoproteome after simulated MAPK activation. Based on their gene ontology (GO) classifications (Figure 6D), putative phosphoproteins from the 4–5 h time points are enriched for functions in “transport mechanisms”—in particular vesicle-mediated and nucleocytoplasmic transport. This is in agreement with rapid movement of activated MAPKs into the nucleus (Ligterink et al., 1997; Lee et al., 2004) and observed accumulation of secreted defense metabolites (Figure 2). This is followed (starting at 7 h) by proteins with functions known to be regulated by MAPKs, such as embryo development, stress and immune responses. The “later” proteins from the 7–8 h time points appear to be responsible for supplying the biosynthetic precursors and the energetic requirement for production of the numerous defense secondary metabolites. The appearance of components of vacuolar transport and catabolic processes (at 7–8 h) also corroborate the eventual onset of cell death.

Overall, the above analysis revealed a phosphoproteome reprogramming through MPK3/6 activation. These phosphoproteins (consisting of both direct MAPK substrates and other “indirect” phosphoproteins) for the 4–8 h time points are listed in Figures S10–S13. To facilitate an initial distinction of potential substrates, all phosphoproteins lacking the typical “S/T-P” MAPK-targeted phosphosites are marked in gray in the tables and number of “S/T-P” sites in the putative MAPK targets is indicated. A selection of phosphoproteins with putative functions in plant defense is listed in Table 1, which includes known MAPK substrates. Examples are the Tandem Zinc Finger 7 (TZF7, previously identified as an *in vitro* MPK3/6 substrate; Feilner et al., 2005), Nitrate Reductase 2 (NR2, an enzyme required for nitric oxide production; Wang et al., 2010), WRKY33 (a transcription factor involved in camalexin production; Mao et al., 2011) or MVQ1 (a VQ-motif-containing protein recently shown to control WRKY-regulated defense gene expression; Pecher et al., 2014). In addition, several WRKYs and gluthathione-*S*-transferases (GSTs) were also found to be putative MAPK substrates.

**Table 1:** **Selected putative MAPK substrates and phosphoproteins with potential roles in plant defense**.

**AGI code**	**Description (alt. names)**	**Fold change(between Col-0 DD and Col-0 KR plants)^a^ (ANOVA^b^/Conf score^c^)**			
		**4 h**	**5 h^d^**	**7 h**	**8 h**
AT1G28280	VQ motif-containing protein (VQ4 or MVQ1)	19.4	2.9	14.0	10.9
		(6.33E-08/124)	(7.24E-07/122)	(1.22E-04/174)	(1.33E-05/311)
AT2G38470	WRKY33	4.6	–	4.2	9.5
		(1.56E-04/161)	(−/−)	(1.32E-07/423)	(3.83E-06/469)
AT1G13960	WRKY4	3.1	–	2.2	3.5
		(5.56E-04/128)	(−/−)	(2.66E-03/26)	(1.33E-03/27)
AT1G62300	WRKY6	23.7	–	38.0	10.8
		(1.82E-03/91)	(−/−)	(4.63E-06/125)	(2.99E-04/279)
AT5G24110	WRKY30	98.5	20.8	70.6	9.5
		(1.19E-06/63)	(7.82E-06/94)	(1.64E-11/118)	(5.37E-06/267)
AT1G80840	WRKY40	29.8	–	–	12.9
		(2.55E-05/34)	(−/−)	(−/−)	(1.21E-06/65)
AT3G11820	PEN1 (SYP121)	–	–	–	2.1
		(−/−)	(−/−)	(−/−)	(2.14E-02/25)
AT2G44490	PEN2 (BGLU26)	–	–	5.3	–
		(−/−)	(−/−)	(1.68E-02/32)	(−/−)
AT1G59870	PEN3 (PDR8, PDR8)	0.4	–	2.5	3.3
		(7.76E-03/41)	(−/−)	(1.76E-04/625)	(8.25E-05/937)
AT5G44070	PCS1 (PEN4, CAD1, ARA8)	–	–	2.3	2.4
		(−/−)	(−/−)	(1.49E-04/72)	(2.70E-02/24)
AT1G02930	GSTF6 (GST1, ERD11)	2.8	–	–	16.7
		(1.83E-06/37)	(−/−)	(−/−)	(6.16E-06/419)
AT4G02520	GSTF2 (ATPM24)	–	–	2.2	2.8
		(−/−)	(−/−)	(3.51E-03/123)	(1.29E-03/236)
AT1G02920	GSTF7 (GST11, GSTF8)	–	–	18.0	8.9
		(−/−)	(−/−)	(1.08E-12/136)	(7.43E-05/345)
AT3G26830	CYP71B15 (PAD3)	–	–	–	9.7
		(−/−)	(−/−)	(−/−)	(3.65E-06/95)
AT2G30770	CYP71A13	–	–	–	3.4
		(−/−)	(−/−)	(−/−)	(2.26E-03/109)
AT2G41900	Tandem Zinc Finger7, TZF7 (or Oxidative Stress 2, OXS2)	2.8	–	–	2.9
		(4.74E-05/102)	(−/−)	(−/−)	(9.74E-03/84)
AT1G37130	Nitrate reductase2, NR2 (NIA2)	2.6	0.4	0.4	3.4
		(5.68E-04/85)	(6.47E-03/654)	(2.14E-02/338)	(4.99E-03/1113)

a *Putative phosphoproteins enriched using the PAPE method were compared between Col-0 plants expressing the active MKK5-DD and those expressing the inactive MKK5-KR transgene (at the indicated time points after DEX treatment). Only proteins with >2-fold change (p < 0.05, Progenesis software, Nonlinear Dynamics) in at least one of the four time points are shown*.

b *p-value for fold change*.

c *Confidence score for protein ID*.

d *Note that the fold changes from the “5h” samples are not directly comparable to other time points. The “5h” samples were the first batch to be measured, after which the LC-MS system was upgraded in order to increase measurement sensitivity, which apparently affected the relative quantification*.

Interestingly, besides the already mentioned WRKY33 transcription factor, we also find the cytochrome P450 enzymes, CYP71A13 or CYP71B15 (also known as PAD3), as well as members of the non-host penetration resistance pathway, PEN1 to PEN4 (also known as PCS1, Phytochelatin Synthase 1) (Gigolashvili and Kopriva, 2014) amongst the enriched putative phosphoproteins. Thus, the increased presence of these enzymes/proteins (presumably in their phosphorylated forms) correlates with the observed accumulation of camalexin and glucosinolate-derivatives (in Figure 2), which supports the notion of increased biosynthesis. Taken together, we could enrich for a number of putative phosphoproteins after an artificial activation of MPK3/6. Since some of these contain typical MAPK-targeted phosphosites, they represent potential direct MAPK substrates.

### Validation of MAPK substrates

**Figure 7:**
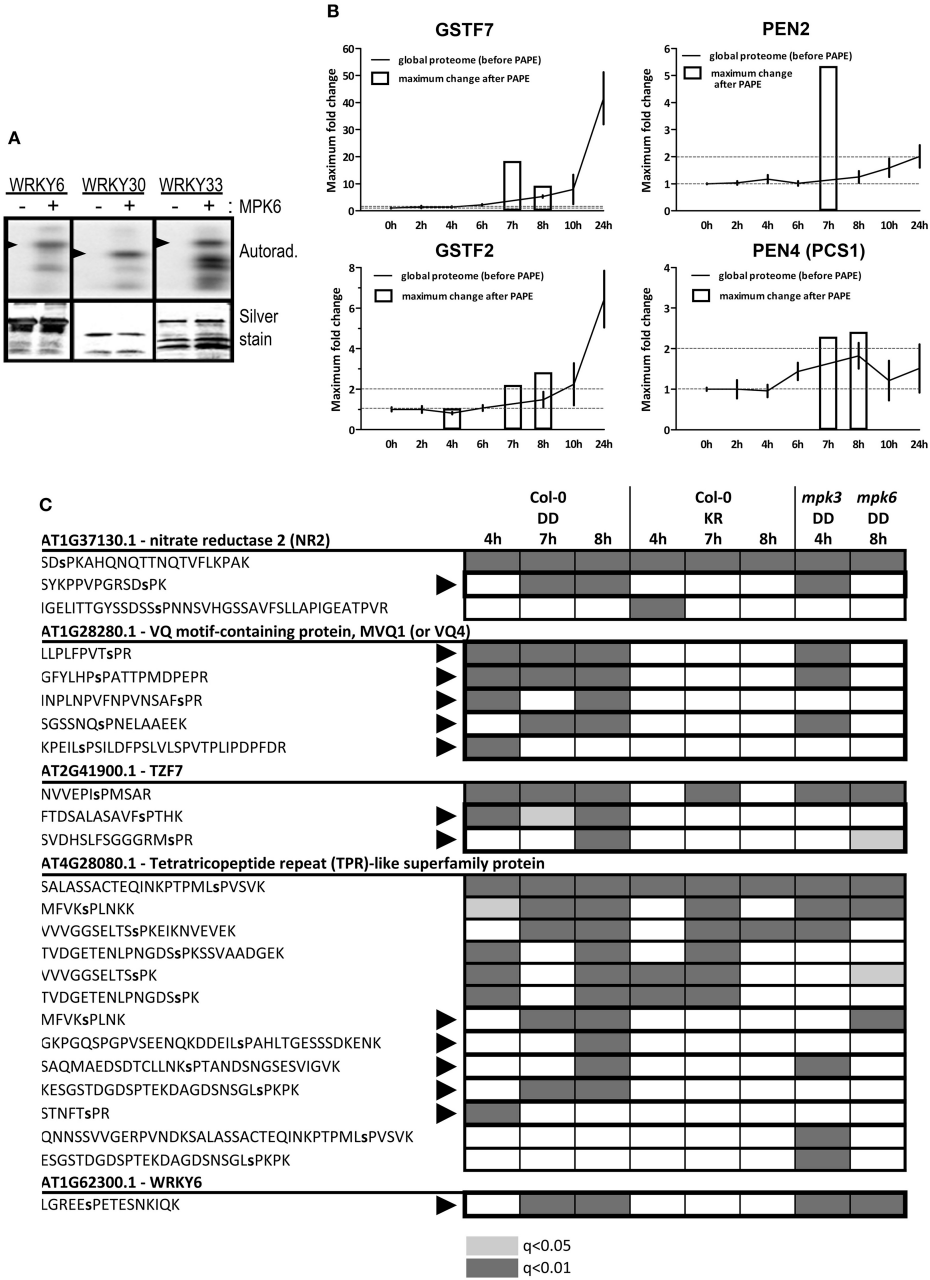
**Validation of phosphorylation of the identified putative substrates. (A)** *In vitro* kinase assay using recombinant MPK6 and recombinant His-tagged WRKY proteins as substrates in the presence of γ−^32^P-ATP. Phosphorylated substrates are visualized by phosphor-imaging (top panel) and equal loading determined by silver staining of the gels (bottom panel). Arrowheads mark the position for the expected size of the full-length proteins (smaller protein bands are presumably from partial or degradation products). **(B)** Comparison of protein levels before and after the PAPE procedure for selected putative substrates/phospho-proteins. The relative fold phospho-protein enrichment after PAPE (between the Col-0 DD and the Col-0 KR plants) for the 4, 7, and 8 h time points after DEX treatment (white bars) were superimposed over the protein levels detected over the 24 h global proteome analysis. **(C)** Phosphorylation of SP/TP motifs within selected putative MAPK substrates. Proteome Discoverer 1.4 was used to consolidate the data from different experiments. The shadings denote the detection of the indicated phosphopeptides (gray: *q* < 0.01 or light gray: *q* < 0.05). Black arrowheads mark the phosphopeptides that are only detected in the Col-0 DD but not the KR lines. The phosphorylated residues are indicated as small letters within the peptide sequence.

A thorough validation through independent phosphorylation assays would be required to differentiate between the direct MPK3/6 targets and those “indirect” phospho-targets arising from increased protein abundance or through the actions of other kinases activated downstream of the MAPKs. As proof-of-principle, we selected a few candidates and could confirm that WRKY6, WRKY30 and WRKY33 were indeed used as substrates by MPK6 in an *in vitro* kinase reaction (Figure 7A).

Another simple but indirect assessment of whether a protein can be potentially phosphorylated is to compare its abundance profile before and after the PAPE step. For instance, in contrast to GSTF2, the enrichment after the PAPE procedure for GSTF7 (4–8 h) does not mirror its overall expression profile (0–24 h). Similarly, levels of PEN2 and PEN4/PCS1 appear to be constant or only slightly changed over the 24 h period but are detected in the phosphoprotein enriched fractions at 7 and 7–8 h, respectively (Figure 7B). This would support the notion that these proteins were indeed phosphorylated (albeit not necessarily by MPK3 or MPK6 directly). A weakness of this method is the requirement of detection in the total proteome analysis but this is often challenging if these proteins are of insufficient abundance in the crude extracts.

Obviously, the most direct evidence would be the identification of the phosphopeptides that match typical MAPK targeted phosphosites. We consolidated all the phosphopeptides identified in all the PAPE samples, which resulted in in 1220 high confidence phosphopeptides (Proteome Discoverer 1.4, false discovery rate adjusted *q* < 0.05) and another 1600 putative phosphopeptides (*q* < 0.05 but >0.01) (Table S16) and filtered for phosphopeptides with typical MAPK-targeted SP or TP residues. This revealed 62, 17, 27, and 137 peptides with phosphorylated SP or TP residues for the 4–8 h samples, respectively (Table S17). For illustration, five putative MAPK substrates and their associated phosphopeptides are shown (Figure 7C). Some phosphopeptides were only detected in the Col-0 DD plants but not the Col-0 KR plants, suggesting these are phosphorylated after MPK3/6 activation (e.g., MVQ1, TZF7, and WRKY6). A few other phosphopeptides pre-existed in selected time points of the Col-0 KR plants, which meant they were already phosphorylated prior to the MPK3/6 activation (e.g., one phosphopeptide for NR2 or some phosphopeptides in the tetratricopeptide repeat (TPR)-like protein encoded by *At4g28080*, Figure 7C). In addition, we also checked for these phosphopeptides in plants with the MKK5^DD^ transgene in* mpk3* or *mpk6* background. The similar or differential detection of these phosphopeptides in these mutant backgrounds indicate either redundancies between the two MAPKs or preferential dependence on one of the two MAPKs in mediating the phosphorylation, respectively (Figure 7C). For instance, detection of MVQ1 phosphopeptides is absent in the *mpk6* background (Figure 7C), which is in agreement with recent data from our laboratory that MPK6 is the major *in vivo* kinase for MVQ1 after MAMP elicitation (Pecher et al., 2014).

To validate the detected phosphosites, we also performed a targeted proteomics analysis where an “inclusion list” of the desired phosphopeptides (Table S20) was used to enable relative quantification of the phosphosites within each protein. For 17 of these putative substrates, the phosphopeptides with higher peptide spectral match (PSM) scores mark the relatively most abundant *in vivo* phosphosites (Table 2). For instance, although previously shown to be multiply phosphorylated at nearly all its potential phosphosites (Pecher et al., 2014), the S235 and S215 residues in the C-terminus of the MVQ1 protein appear to be the most prominent *in vivo* phosphosites (Table 2). Similarly, the predominant *in vivo* phosphosites could be identified for three known *in vitro* MPK3/6 substrates (TZF7, WRKY6, and NR2), as well as 13 other novel MPK3/6 targets using the targeted proteomics approach (Table 2). In summary, our phosphoproteomics approach enabled several known and numerous novel candidate MAPK substrates that are potentially involved in defense regulation to be identified.

**Table 2:** **Identified phosphorylation sites on selected putative substrates**.

**Accession**	**Description**	**Phosphopeptide^a^**	**MH+ [Da]**	**PSM score^b^**	**Modifications**	**pRS Score**	**IonScore control**	**IonScore 4 h**	**IonScore 5 h**	**IonScore 7 h**	**IonScore 8 h**
AT4G38550	PEARLI 4 family	NSSPPsPLHPAASHsPPPPQPYR	2578.12296	2852	S6; S15	58	–	32		43	56
		NSSPPSPLHPAASHsPPPPQPYR	2498.15128	777	S15	49	–			42	65
		stPGSPAHPPGARSPPPSYLSNK	2462.08756	669	S1; T2	52	–	27		31	34
		NSsPPsPFHPAAYK	1659.65691	199	S3; S6	92	–				44
		SNHGKEQIEDFYEQDDDVtPR	2602.06693	171	T19	134	–				83
		MEAMSYEPETNAPSsPYHPAGNR	2616.05472	60	S15	81	–				52
AT4G28080	Tetratricopeptide repeat	SALASSACTEQINKPTPMLsPVSVK	2696.30051	833	S20	68	–	55		65	72
		VVVGGSELTSsPKEIKNVEVEK	2408.22928	492	S11	39	–				62
		TVDGETENLPNGDSsPKSSVAADGEK	2684.15677	129	S14	73	–	30			64
		MFVKsPLNKK	1271.65830	55	S5	75	–	26		28	30
		MFVKsPLNK	1143.56120	6	S5	104	–				30
AT1G28280	VQ motif-containing protein	LLPLFPVTsPR	1319.71318	1657	S9	129	–	45		45	49
	(VQ4 or MVQ1)	GFYLHPsPATTPMDPEPR	2092.91086	769	S7	108	–	46		34	47
		INPLNPVFNPVNSAFsPR	2063.01714	362	S16	167	–	62	85		87
		SGSSNQsPNELAAEEK	1727.70293	168	S7	119	–			67	54
AT2G41900	CCCH-type zinc finger protein 7	FTDSALASAVFsPTHK	1758.81168	172	S12	86	–	40			44
	(TZF7), or oxidative stress 2	NVVEPIsPMSAR	1379.63786	93	S7	77	–	25		15	35
	(OXS2)	SLSSRELRTNSsPIVGSPVNNNTWSSK	2997.43960	6	S12	14	–				16
AT4G11740	Ubiquitin-like superfamily protein	SRSGsPEEEHASINPAER	2112.83567	116	S3; S5	77	–			14	29
AT1G79280	Nuclear pore anchor	VPSSTPLIKsPVATTQQLPK	2172.16605	42	S10	50	–			28	
		SPEKEEVQPETLATPTQsPSR	2391.10508	20	S18	103	–				29
AT4G33400	Vacuolar import/degradation	LRPKsPSSSLDDVEAK	1808.87546	41	S5	83	–	24			38
AT5G53440	Unknown protein	DGRRsPDYQDYQDVITGSR	2307.99460	10	S5	74	–	40			
AT4G17720	RNA-binding family protein	VHLSEsPKAASSTQEAERESK	2351.08554	577	S6	98	–	37		54	54
AT1G07110	Fructose-2,6-bisphosphatase	GLFVDRGVGsPR	1339.65012	12	S10	38	–			18	
AT1G37130	Nitrate reductase 2 (NR2)	SYKPPVPGRSDsPK	1594.76189	310	S12	89	–			22	28
AT1G62300	WRKY6	LGREEsPETESNKIQK	1924.89969	446	S6	98	–			41	40
AT2G26570	WEB1 (weak chloroplast movement under blue light 1)	FSGSPVSTGtPKNVDSHR	2032.85007	122	T8; T10	51	–			35	24
AT1G59710	Unknown protein	FFRQESTDSLAVGsPPKSEGR	2375.10093	322	S14	82	–			47	35
AT2G20960	PEARLI 4 family	RSNTSDTRPRtPIHESAATGR	2390.13466	106	T11	49	–			20	34
AT2G20950	PEARLI 4 family	NRPSSPFHPSQSRsPPPHAR	2399.04946	211	S4; S14	35	–			24	23
AT4G38710	Glycine-rich protein	TLPVAVVEVVKPEsPVLVIVEKPK	2649.52494	49	S14	44	–				58

a *Small letters indicate phosphorylation of the Ser or Thr residue (phosphorylated residues typical for MAPK-targeted sites are further underlined)*.

b *PSM, peptide spectral match*.

## Discussion

### Sustained MAPK activation is sufficient to drive antimicrobial metabolite accumulation

In this work, we “hijacked” the MAPK pathway by introducing a constitutively-active MKK5 to mimic pathogen- or stress-mediated MPK3/6 activation. Since MPK3 and MPK6 are also involved in various developmental processes such as leaf abscission and development of stomata, anthers or embryos (Wang et al., 2007; Cho et al., 2008; Hord et al., 2008; Wang et al., 2008), such an artificial MAPK activation may lack proper regulation and one may question its physiological relevance. However, our system is focused on a specific developmental stage and tissue (i.e., 6-week-old leaves) within a defined time period. Hence, the effects of MPK3/6 activation for the above-mentioned developmental processes are of little bearing in this system. In contrast to the typical transient MAPK activation after MAMP treatment (typically lasting for less than 60 min), the use of the dexamethasone induction here causes a prolonged MPK3/6 activation. Our metabolite profiling showed that such sustained MPK3/6 activation elicited a massive reprogramming of the defense metabolome, where we found high accumulation of camalexin, indole glucosinolate derivatives (Figure 2, Figures S2A–C). Additionally, levels for many defense-related phytohormones such as JA, SA (Figures S2G,H), and ET (Han et al., 2010) were also elevated (albeit tissue damage may account for accumulation of some of the “late” metabolites). By contrast, flg22 elicitation, which causes only transient MAPK activation, led to accumulation of comparatively low levels of camalexin (Millet et al., 2010; Schenke et al., 2011). Thus, the system used is comparable to the extended MAPK activation effected during effector-triggered immunity as compared to PTI. Recent work also suggested that sustained MAPK activation is “a critical determinant for modulation of robustness of the immune signaling network” (Tsuda et al., 2013). A conclusion of the metabolome analysis presented here is that sustained MAPK activation is sufficient, and apparently the key determinant, for reprogramming cellular metabolic pathways into antimicrobial and defense metabolite production.

### Role of ROS from RBOHD for defense metabolite accumulation and cell death

Manipulating *in vivo* phytoalexin production can be a way to engineer pathogen-resistant plants. However, the sustained MAPK activation also caused cell death that would be counterproductive for plant growth and crop yield (Figure 1). Since ultimate cell death was partially reduced in the *ein3/eil1* and *rbohD* mutants (Figure 1), we compared the observed phenotypes in the different tested genotypes. Overall, MPK6 appears to be more important than MPK3 in mediating accumulation of the tested defense metabolites (Figures 3A–C). While acetylagmatine required either ethylene or ROS generated by RBOHD (Figure 3C), accumulation of the Trp-derived antimicrobial substances are independent of ethylene signaling (Figures 3A,B). RBOHD is, however, required for camalexin accumulation. The related RBOHF was previously thought to play a stronger role than RBOHD for camalexin accumulation induced by introducing a mutated catalase allele (Chaouch et al., 2012). Although we did not investigate RBOHF directly, RBOHF activity is still present in the *rbohD* mutant used here and therefore suggests RBOHD activity (instead of RBOHF) being essential for camalexin and acetylagmatine accumulation. Our findings are therefore in agreement that RBOHD is the major contributor for MAMP-induced ROS (Mersmann et al., 2010; Ranf et al., 2011). A conflict with previous studies is that the ROS generated in such chemically-induced MAPK plants has been reported to be of chloroplastic origin (Liu et al., 2007). Interestingly, while cell death induction is seen in all tested genotypes, new rosette leaves emerged from the apical meristem of the *rbohD* plants (Figure 1), indicating that ROS is needed for complete cell death. Thus, besides chloroplastic ROS, RBOHD-generated superoxide is also important. Cell-to-cell propagation and amplification of ROS mediated by RBOHD (Miller et al., 2009; Dubiella et al., 2013) could account for the penetrance of cell death induction into the deeper meristem tissues. Since this ROS propagation circuit is mediated by RBOHD phosphorylation through the calcium-dependent protein kinase, CPK5 (Dubiella et al., 2013), it raises the question if the elevated MAPK activities in our system may crosstalk with the CPK5 pathway. Taken together, accumulation of defense compounds and cell death are intertwined and further studies are needed to separate these two processes in order to exploit this knowledge for increasing plant resistance without adverse penalties for crop yield.

### Differences between MPK3 and MPK6

The aim of this study is to identify downstream phospho-targets (including direct substrates) of MPK3 or MPK6. To identify specific MPK3 or MPK6 substrates, we also compared the PAPE-fractionated phosphoproteins from *mpk3* and *mpk6* backgrounds to the “core” set of experiments based on the Col-0 genotype. A single time point was chosen for this analysis. Due to slightly variable expression profiles of the transgene in the different genotypes, we decided on 4 h (*mpk3*) or 8 h (*mpk6*) after DEX treatment for comparison to the Col-0 background. This timing was based on a pilot study of the induction profile of the MKK5^DD^-expression in the different genotypes. Comparison of the resulting protein lists showed that most of the enriched phosphoproteins in Col-0 were reduced in levels or missing in the *mpk6* background (Figure S3, Table S15), suggesting it is the major kinase for the differentially detected phosphoproteins. Specific examples include WRKY6, WRKY30, WRKY33, and WRKY40 (Figure S3B). By contrast, abundance of putatively phosphorylated WRKY3, WRKY6, and WRKY33 (at 4 h post DEX) in the *mpk3* mutant is mostly like in Col-0 plants (Figure S3A). Despite this apparent reliance on MPK6 activity, the *mpk3* mutant also had many unique putative phosphoproteins (i.e., more abundantly detected in the *mpk3* mutant compared to Col-0; see lower half of Table S15A). Taken together with the global proteome data that a cluster of proteins involved in translation is missing in the *mpk3* mutant (Figure 5), as well as the previously reported haploinsufficiency of MPK3 in ovule development (Wang et al., 2008), there is differential preference for the various substrates by the two MAPKs, and they are not totally redundant as often thought. A caveat of the above phosphoproteomics comparative analysis is that only a single timepoint was chosen; future studies should preferably include a time course series (as done for the “core” experiment) to allow for variance in induction profiles.

### Comparison to other proteomics studies of MAPK substrate identification

In a similar analysis to uncover candidate MPK3/6 substrates, metal-oxide affinity chromatography was used to enrich for phosphopeptides in Arabidopsis expressing the tobacco MKK5^DD^ variant (Hoehenwarter et al., 2012). Besides the origin of the MKK5 construct, another difference to this work is the tissue type and developmental stage employed: they used 12-day-old seedlings liquid-grown in continuous light while our plants are 6-week-old adult soil-grown plants under standard short day light regime. Potential substrates required for rapid response to pathogen attack are presumably pre-existing in leaves as hypo-phosphorylated forms. We may assume that using adult leaves bears more potential in finding substrates relevant for the pathogen response under natural growth conditions. Furthermore, Hoehenwarter et al. (2012) compared seedlings with or without DEX treatment while we used the expression of a kinase-inactive MKK5^KR^ variant to exclude any impact arising from the expression of the MKK5 protein alone. Their list of candidate MPK3/6 substrates contains 140 proteins. Our consolidated list from the four time points (4–8 h) yielded 539 putative phosphoproteins (Table S14). Thirty-seven of these proteins are common between our list and that of Hoehenwarter et al. (2012) (Figure S4, Table S18), which includes known MPK3/6 substrates such as TZF7, NR3, MVQ1 (VQ4), and WRKY40 (Feilner et al., 2005; Popescu et al., 2009; Wang et al., 2010; Pecher et al., 2014). The MAPK phosphatase, MKP1, a reported substrate of MAPKs (Gonzalez Besteiro and Ulm, 2013) and the universal stress proteins PHOS32/34 (Merkouropoulos et al., 2008) are found in the 103 candidates unique to the “Hoehenwarter” list. Similarly, in the candidates found exclusively in our list are also known MAPK substrates such as the chromatin remodeling ATPase, BRAHMA (Feilner et al., 2005), decapping complex protein1, DCP1 (Xu and Chua, 2012), WRKY6 (Popescu et al., 2009), and WRKY33 (Mao et al., 2011) (Figure S4). Thus, both studies enabled *bona fide* MAPK substrates to be identified; but the differences in the studies also highlight that each study has its strengths or weaknesses.

One of the strength of the Hoehenwarter et al. (2012) work is the direct detection of the SP or TP residues in the phosphopeptides. We were also able to identify many phosphopeptides with phosphorylated SP or TP residues (Table S17) but for other candidates, direct detection of such sites were not found. The latter is likely due to the fact that our work is based on phosphoprotein enrichment and it is difficult to detect low abundance phosphopeptides in the complex peptide mixtures reintroduced after tryptic digest of the proteins. One consideration for the future is to add another phosphopeptide enrichment step after the PAPE procedure to enhance phosphopeptide detection sensitivity. Nevertheless, the advantage of our analysis is that our list yielded a larger number of candidates for future analysis. Of course, the larger number of candidates we obtained may also be accounted by the use of a range of time points for the DEX treatment, which may cover early and late substrates. Since some MAPK substrates are destabilized upon phosphorylation (e.g., MVQ1, Pecher et al., 2014), their detection in the current experimental set-up is a balance between the rate of protein decay and efficiency of the phosphoprotein enrichment. In fact, we observed some fluctuations within the 4–8 h time course in abundance of some of the candidates (Table S14) or the detection of specific phosphopeptides (an example is At4g28080, see Figure 7B). This may reflect a change in the protein destabilization rate and therefore usage of several time points enhances the detection window and improves the chance of discovery. In this respect, one should also consider the candidate phosphoproteins that are down-regulated in the MKK5^DD^ line compared to the MKK5^KR^ line (Figure 6B, Tables S10–S13).

### Identification of known and potential novel MAPK substrates

Identification of known MAPK substrates in the consolidated list of the enriched phosphoproteins (4-8 h after DEX treatment), such as MVQ1, NR2, TZF7, VIP1, and several WRKYs (Feilner et al., 2005; Djamei et al., 2007; Wang et al., 2010; Mao et al., 2011; Maldonado-Bonilla et al., 2014; Pecher et al., 2014), confirmed that the artificial MAPK activation system coupled to an efficient phosphoprotein enrichment method (Lassowskat et al., 2013) can identify direct MPK3/6 substrates that regulate plant immunity responses. Among the potential novel substrates and downstream “indirect” phosphoproteins, we want to particularly highlight three functional groups that deserve future attention:

- The first is a group of processing-body (P-body) components such as the mRNA decapping complex proteins, DCP1 and VARICOSE (VCS) and the exoribonuclease, XRN4 (Xu and Chua, 2010, 2012). We also found TZF7 as an enriched putative phosphoprotein. Recently, we showed that the related TZF9 is an MPK3/6 substrate and colocalizes to P-bodies (Maldonado-Bonilla et al., 2014). Remarkably, the putative RNA ligase/cyclic nucleotide phosphodiesterase family protein, encoded by At3g28140, which was found as an interacting partner of both TZF7 and TZF9 in a large-scale interactome study (Arabidopsis Interactome Mapping, 2011), was also phospho-enriched in the 7 h sample (Table S14). Taken together with the report on MPK6 phosphorylation of DCP1 (Xu and Chua, 2012), there is rising evidence that MAPKs affect post-transcriptional gene regulation through elements of mRNA processing in P-bodies.
- The second group includes proteins such as exocyst components (Exo70B2, Exo70E1, Sec3A, Sec5A), COPII components of ER-associated vesicles (Sec23/Sec24), the Sec7 guanine exchange factor, the SecY translocon, or several clathrin-related proteins. These proteins are involved in vesicle/membrane transport or secretion, and therefore may mediate the observed secretion of defense metabolites and presumably shuttling of key defense proteins in various membrane compartments.
- A third group of proteins are those that may be responsible for the indole glucosinolate and camalexin accumulation. PAD3 is a camalexin biosynthetic enzyme, whose expression is regulated by Mao et al. (2011). The detection of the PEN1-PEN4 proteins around 7–8 h after DEX treatment (Table 1) is just slightly trailing behind the increase of various indole glucosinolate derivatives (typically <6 h, Figure 2). Nevertheless, the rapid increase in the indole glucosinolate metabolites is indicative for increased activities of these PEN proteins. Although PEN1, PEN3, and PEN4/PCS1 are known phosphoproteins (Nühse et al., 2003; Stein et al., 2006; Benschop et al., 2007; Wang et al., 2009), many of the reported phosphosites are not the typical MAPK targeted sites; thus it is unclear if these are direct MAPK targets or detected due to increased protein abundance. In the case of PEN2, it was highly phospho-enriched (at 7 h after DEX treatment) although there was no apparent increase in expression in the global proteome analysis (see comparison in Figure 7B). Hence, PEN2 is a candidate phospho-target after MAPK activation. This raises the possibility that activities of PEN proteins are regulated by phosphorylation through MAPKs or other kinases, which must be tested in the future.

## Concluding remarks

In conclusion, this work provided a catalog of potential phosphoproteins downstream of MPK3/6 activation, including direct MPK3/6 substrate candidates. The importance of sustained MPK3/6 activation in plant chemical defense regulation is uncovered through a parallel metabolomics analysis that revealed a massive reprogramming of defense metabolism—in particular accumulation of camalexin, indole glucosinolate, agmatine and a variety of their derivatives. Several of the detected phosphoproteins/MAPK substrate candidates are involved in the corresponding regulatory (or biosynthesis) pathways and may account for the production of these defense compounds. Thus, besides re-programming of chemical defense, and as already discussed above, many of the other putative MAPK substrates or “indirect” phosphoproteins are components of RNA metabolism or secretory pathways. Notably, there is so far only limited information on MAPK-mediated control of the latter two processes. This work therefore identifies the putative phosphoproteins as a basis for elucidating the MAPK-mediated regulon of plant defense against pathogens or abiotic stresses. Future work will focus on uncovering the impact of phosphorylation on the activities or function of these phospho-proteins or MAPK substrates.

Dataset: The proteomics data have been deposited to the ProteomeXchange Consortium (Vizcaíno et al., 2014) (http://proteomecentral.proteomexchange.org) via the PRIDE partner repository with the dataset identifier PXD001252 and DOI 10.6019/PXD001252.

### Conflict of interest statement

The authors declare that the research was conducted in the absence of any commercial or financial relationships that could be construed as a potential conflict of interest.
