# Supplementary material for: Sustained mitogen-activated protein kinase activation reprograms defense metabolism and phosphoprotein profile in Arabidopsis thaliana
Source: Front Plant Sci. 2014 Oct 20;5:554. doi: 10.3389/fpls.2014.00554 (PMC4202796; doi:10.3389/fpls.2014.00554)
Supplement: Supplementary file 1 [file Presentation1.PDF]

Supplementary Material

Sustained mitogen-activated protein kinase activation reprograms defense metabolism and phosphoprotein profile in *Arabidopsis thaliana*.

Ines Lassowskat[1], Christoph Böttcher[1,2], Lennart Eschen-Lippold[1], Dierk Scheel[1], Justin Lee[1]*

[1] Leibniz Institute of Plant Biochemistry, Dept. of Stress & Developmental Biology, Weinberg 3, D-06120, Halle/Saale, Germany

[2] Julius Kühn Institute, Federal Research Centre for Cultivated Plants, Institute for Ecological Chemistry, Plant Analysis and Stored Product Protection, Königin-Luise-Str. 19, D-14195, Berlin, Germany.

*** Correspondence:** Justin Lee (ORCID: 0000-0001-8269-7494), Leibniz Institute of Plant Biochemistry, Dept. of Stress & Developmental Biology, Weinberg 3, D-06120, Halle/Saale, Germany.
jlee@ipb-halle.de

4 supplementary figures
and
20 supplementary tables
(Note: Table S3-S20 are in Excel format and contains sub-tables)

1. Supplementary Figures

FIG. S1. A. Activation of MPK3 and MPK6. Six week old plants were sprayed with 20 µM DEX (0.0075% SILWET L-77) to activate MKK5 gene expression. Zero to 24 hours after treatment, leaves were harvested in liquid nitrogen and used for immunodetection with anti-pTEpY to detect phosphorylated forms of the MAPKs, MPK3/6. **B.** Progression of cell death after DEX (shown for *mpk3* DD as a representative line). Photos were taken at the indicated days (d) after DEX treatment.

A.

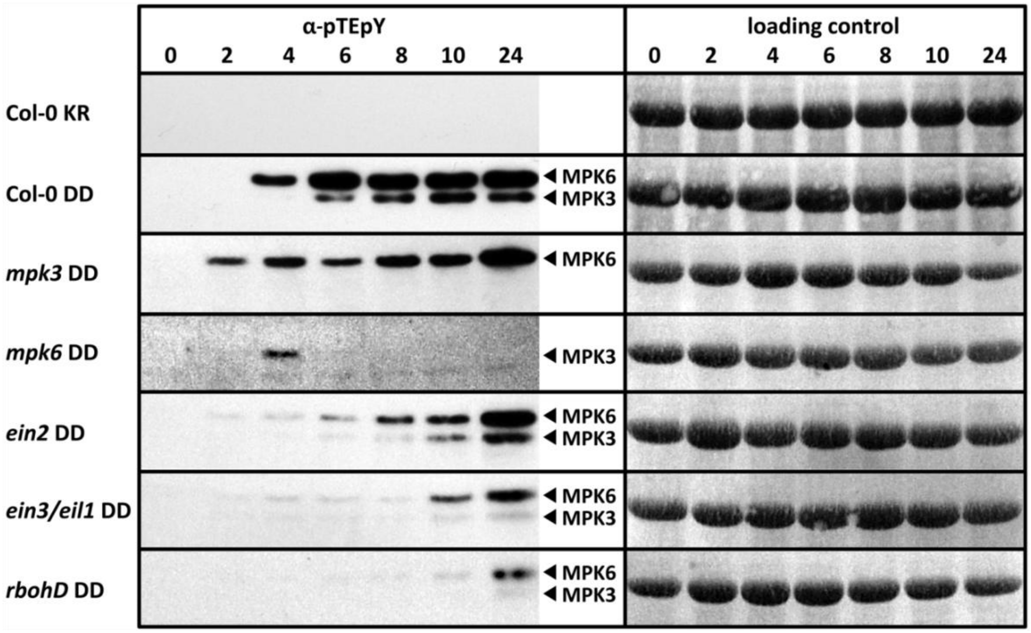

B.

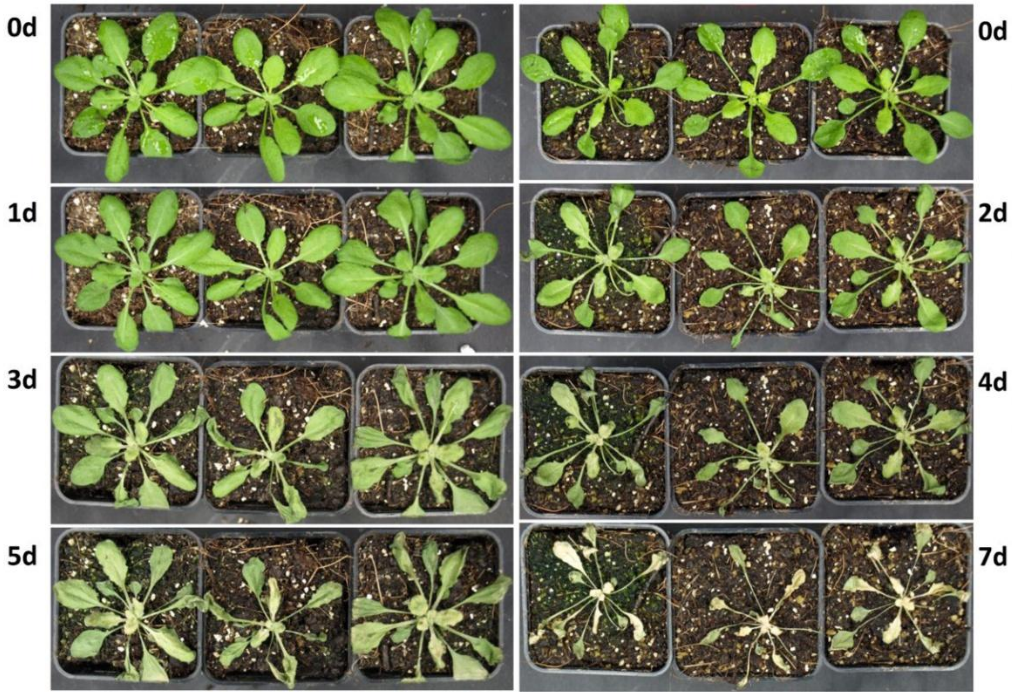

FIG. S2. Additional MPK3/6-triggered metabolic changes. A. Accumulation kinetics of various indole-3-carboxylic acid derivatives. **B.** Accumulation kinetics of Met-derived glucosinolates and corresponding anabolites and catabolites **C.** Accumulation kinetics of known and novel agmatine conjugates (acetylagmatine, phenylacetylagmatine). **D.** Accumulation kinetics of aromatic amino acids and uncommon Phe metabolites (γ-Glu-Phe, Malonyl-Phe, phenylacetyl-conjugates). **E.** Accumulation kinetics of flavonol glycosides. **F.** Accumulation kinetics of ferulate, 5-hydroxyferulate and sinapate esters. **G/H.** Accumulation kinetics of SA, JA and their glucose conjugates. **I.** Chlorophyll degradation. Note that for all the graphs, the y-axis represents the relative abundance of the metabolite (peak area of the quantifier ion) and the x-axis the time (hours, h) after DEX treatment. Error bars represent standard deviations of three independent experiments.

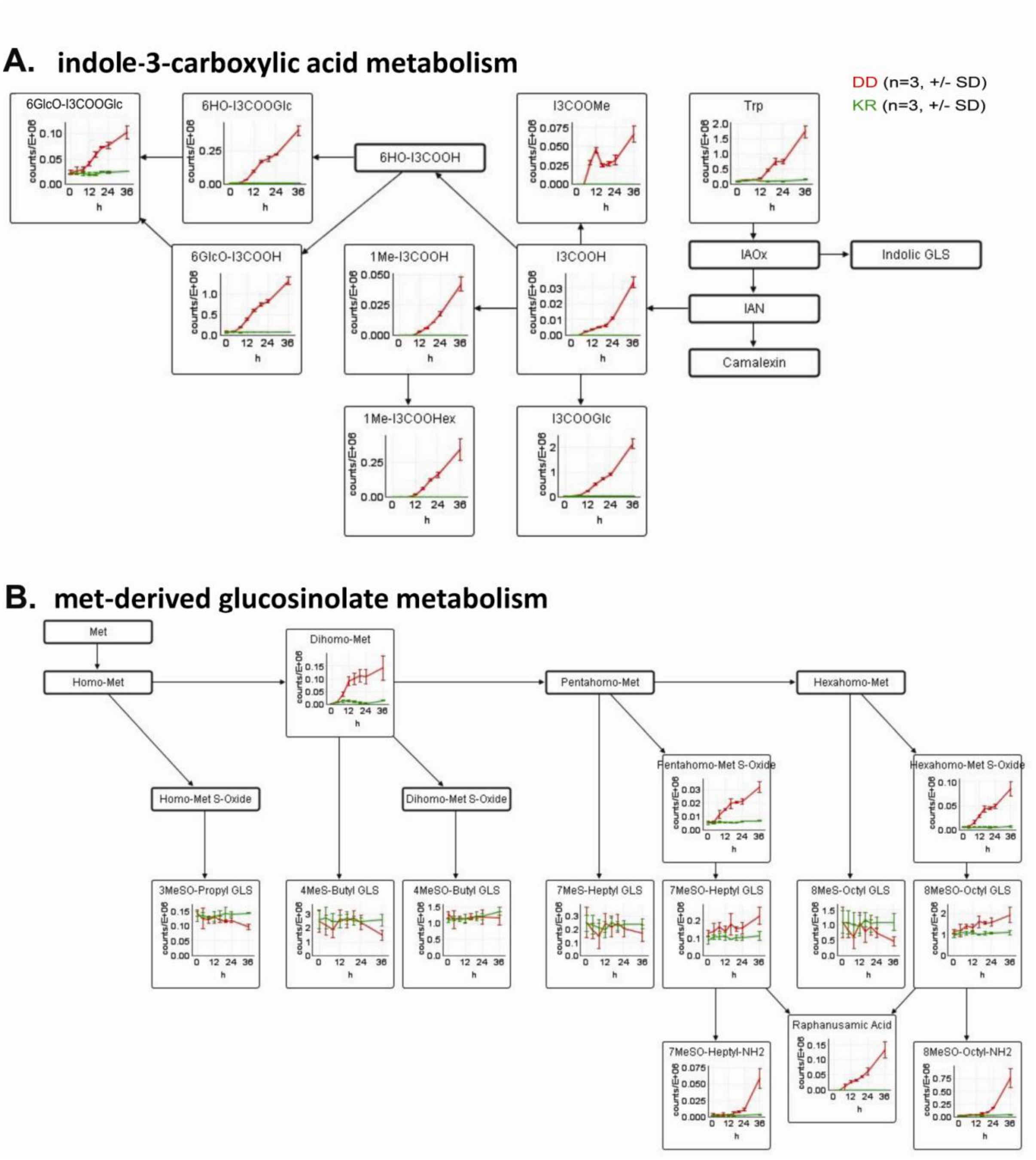

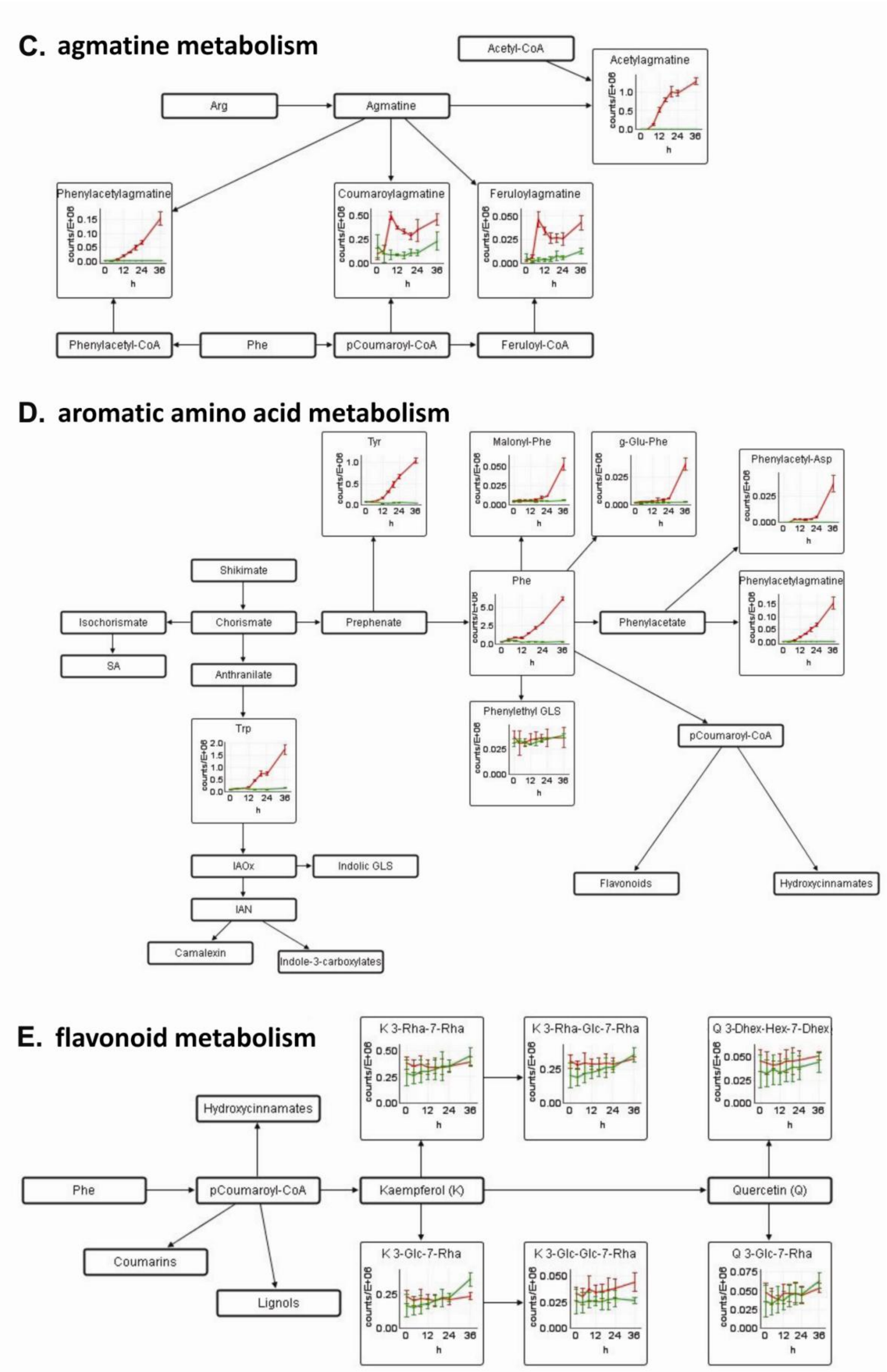
C. agmatine metabolism
Acetyl-CoA
Acetylagmatine
Arg
Agmatine
Phenylacetylagmatine
Coumaroylagmatine
Feruloylagmatine
Phenylacetyl-CoA
Phe
pCoumaroyl-CoA
Feruloyl-CoA
D. aromatic amino acid metabolism
Tyr
Malonyl-Phe
g-Glu-Phe
Phenylacetyl-Asp
Shikimate
Phe
Isochorismate
Chorismate
Prephenate
Phenylacetate
Phenylacetylagmatine
SA
Anthranilate
Trp
Phenylethyl GLS
pCoumaroyl-CoA
IAOx
Indolic GLS
IAN
Camalexin
Indole-3-carboxylates
Flavonoids
Hydroxycinnamates
E. flavonoid metabolism
K 3-Rha-7-Rha
K 3-Rha-Glc-7-Rha
Q 3-Dhex-Hex-7-Dhex
Hydroxycinnamates
Phe
pCoumaroyl-CoA
Kaempferol (K)
Quercetin (Q)
Coumarins
Lignols
K 3-Glc-7-Rha
K 3-Glc-Glc-7-Rha
Q 3-Glc-7-Rha
counts/E+06
h

…

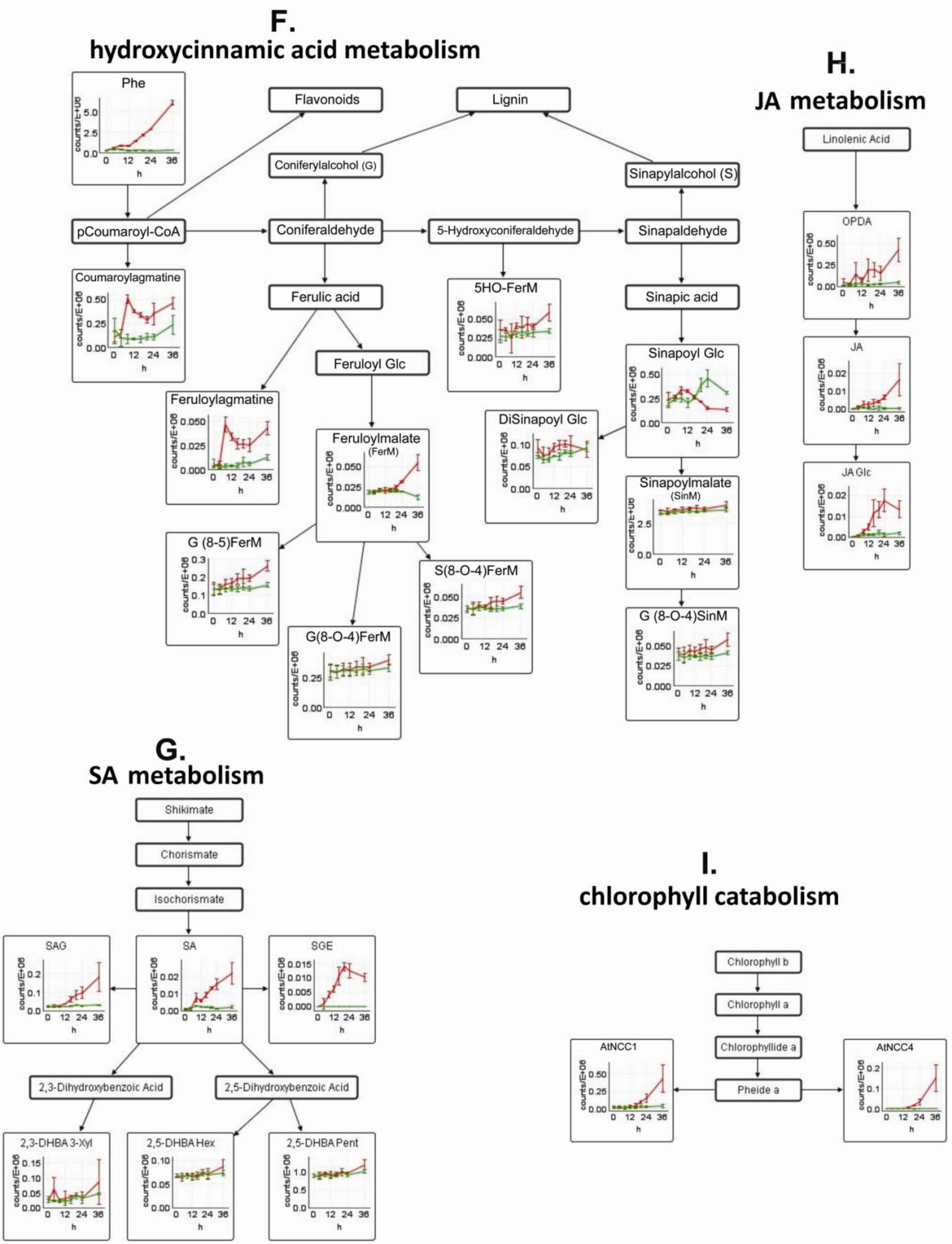
F.
hydroxycinnamic acid metabolism
Phe
Flavonoids
Lignin
Coniferylalcohol (G)
Sinapylalcohol (S)
pCoumaroyl-CoA
Coniferaldehyde
5-Hydroxyconiferaldehyde
Sinapaldehyde
Coumaroylagmatine
Ferulic acid
5HO-FerM
Sinapic acid
Feruloyl Glc
Sinapoyl Glc
Feruloylagmatine
DiSinapoyl Glc
Feruloylmalate
(FerM)
Sinapoylmalate
(SinM)
G (8-5)FerM
S(8-O-4)FerM
G(8-O-4)FerM
G (8-O-4)SinM
H.
JA metabolism
Linolenic Acid
OPDA
JA
JA Glc
G.
SA metabolism
Shikimate
Chorismate
Isochorismate
SAG
SA
SGE
2,3-Dihydroxybenzoic Acid
2,5-Dihydroxybenzoic Acid
2,3-DHBA 3-Xyl
2,5-DHBA Hex
2,5-DHBA Pent
I.
chlorophyll catabolism
Chlorophyll b
Chlorophyll a
Chlorophyllide a
Pheide a
AtNCC1
AtNCC4
counts/E+06
h

FIG. S3. Differential abundance of selected phosphoproteins in *mpk3* DD and *mpk6* DD lines, as compared to the core experiment (Col-0 KR and Col-0 DD lines). Relative abundance of the proteins was normalized to the maximum values detected for each protein (set as 100%). Each bar represents means and standard deviations (n=6). **A.** *mpk3* DD plants were collected 4 h post DEX treatment and compared to data in Table S10; **B.** *mpk6* DD plants were collected 8 h post DEX treatment and compared to data in Table S13.

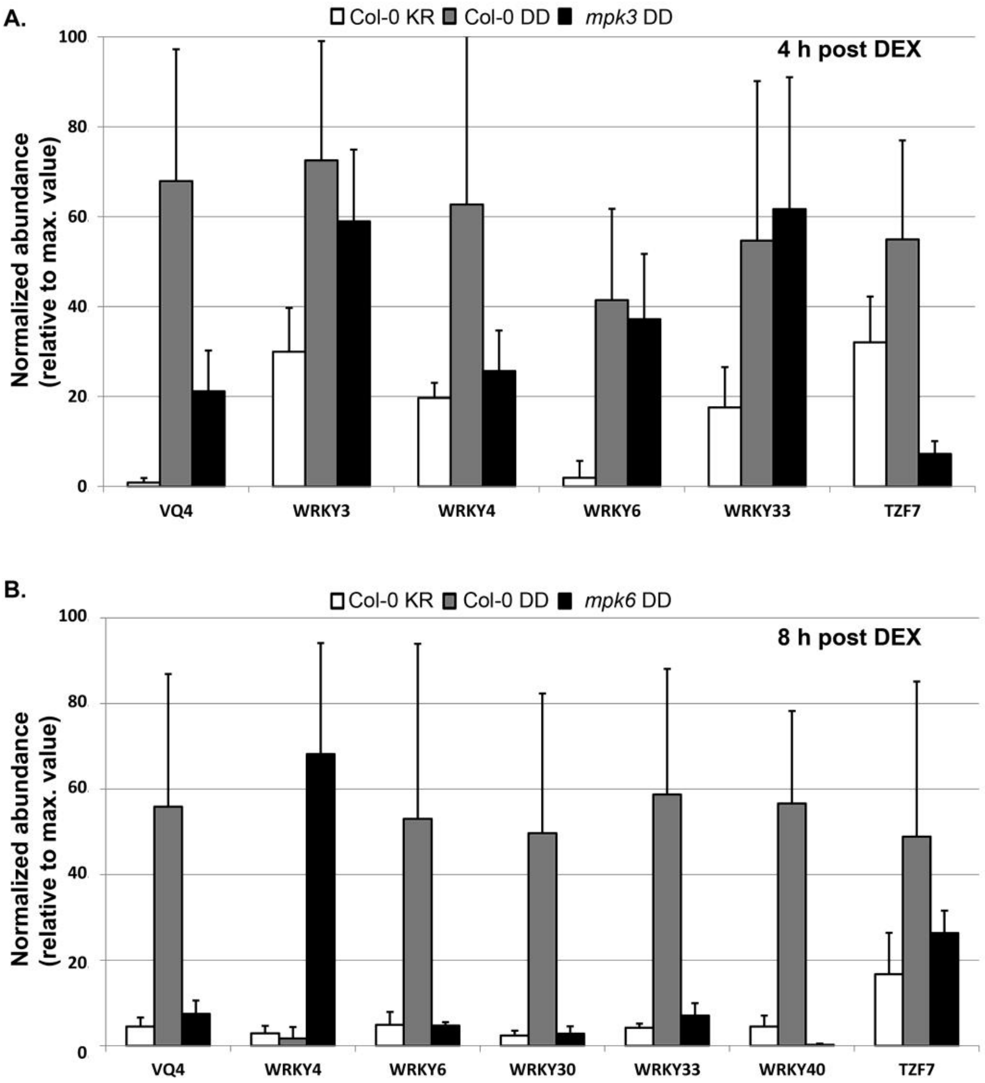

FIG. S4. Venn diagram comparison of candidate phosphoproteins identified in this study to those reported in Hoehenwarter et al. (2012). Proteins listed in Table S14 were compared to the list published by Hoehenwarter *et al.* (2012) and plotted in a Venn diagram. Known substrates of MPK3 or MPK6 are indicated. Detailed list of proteins within each group is listed in Table S18.

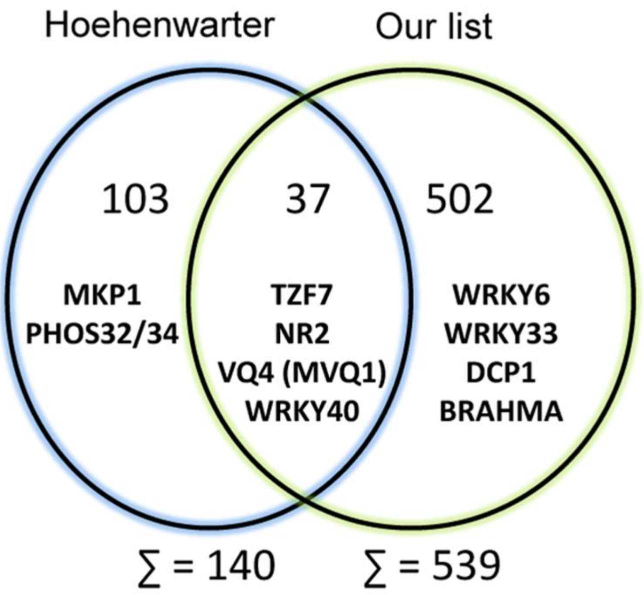

2. Supplementary Tables

SUPP. TAB. S1. Analytical data of annotated compounds

SUPP. TAB. S2. Collision-induced dissociation (CID) mass spectral data of annotated compounds.

(The following supplementary tables are in Excel format and available online)

SUPP. TAB. S3. Differentially regulated proteins in control (KR) 0-24 h after DEX treatment.

SUPP. TAB. S4. Differentially regulated proteins in wild type DD 0-24 h after DEX treatment.

SUPP. TAB. S5. Differentially regulated proteins *mpk3* 0-24 h after DEX treatment.

SUPP. TAB. S6. Differentially regulated proteins in *mpk6* 0-24 h after DEX treatment.

SUPP. TAB. S7. Differentially regulated proteins in *ein2* 0-24 h after DEX treatment.

SUPP. TAB. S8. Differentially regulated proteins in *ein3/eil1* 0-24 h after DEX treatment.

SUPP. TAB. S9. Differentially regulated proteins in *rbohD* 0-24 h after DEX treatment.

SUPP. TAB. S10. Differentially regulated proteins four hours after DEX treatment and PAPE enrichment.

SUPP. TAB. S11. Differentially regulated proteins five hours after DEX treatment and PAPE enrichment.

SUPP. TAB. S12. Differentially regulated proteins seven hours after DEX treatment and PAPE enrichment.

SUPP. TAB. S13. Differentially regulated proteins eight hours after DEX treatment and PAPE enrichment.

SUPP. TAB. S14. Consolidated list of putative phosphoproteins enriched after PAPE procedure (from 4-8 h after DEX)

SUPP. TAB. S15. Differentially regulated proteins after DEX treatment and PAPE enrichment in wild type DD, *mpk3* and *mpk6*.

SUPP. TAB. S16. List of all detected phosphopeptides

SUPP. TAB. S17. List of phosphopeptides with SP or TP motifs

SUPP. TAB.S18. Comparison of our phosphoproteins downstream of MPK3/6 activation to those reported in Hoehenwarter et al (2012).

SUPP. TAB. S19. Relative quantification of annotated compounds by integration of extracted ion chromatograms.

SUPP. TAB. S20. Global Parent Masses used for targeted proteomics.

SUPPLEMENTAL TABLE 1. Analytical data of annotated compounds.

no.	annotation	al[a]	standard used for authentication (source)	lit.[b]	elemental composition	ret. time [s]	quasi-molecular ion				
							type	calculated *m/z*	measured *m/z*	$\lvert\Delta m/z\rvert$[c] *ppm*	mSigma[d]
**1**	Phe	1	Sigma-Aldrich	-	$C_9H_{11}NO_2$	75	$[M+H]^+$	166.0863	166.0859	2.4	13
**2**	*N*-Malonyl-Phe	2	-	-	$C_{12}H_{13}NO_5$	246	$[M+H]^+$	252.0866	252.0854	4.8	32
**3**	γ-Glu-Phe	2	-	-	$C_{14}H_{18}N_2O_5$	179	$[M+H]^+$	295.1288	295.1277	3.7	15
**4**	Tyr	1	Sigma-Aldrich	-	$C_9H_{11}NO_3$	42	$[M+H]^+$	182.0812	182.0812	0.0	5.0
**5**	Trp	1	Sigma-Aldrich	-	$C_{11}H_{12}N_2O_2$	145	$[M+H]^+$	205.0972	205.0961	5.4	5.0
**6**	Leu/Ile	2	Sigma-Aldrich	-	$C_6H_{13}NO_2$	46	$[M+H]^+$	132.1019	132.1014	3.8	9.1
**7**	Glutathione (GS-H)	1	Sigma-Aldrich	-	$C_{10}H_{17}N_3O_6S$	40	$[M+H]^+$	308.0911	308.0891	6.5	33
**8**	Raphanusamic Acid	1	Sigma-Aldrich	[1,2]	$C_4H_5NO_2S_2$	76	$[M-H]^-$	161.9689	161.9679	6.2	12
**9**	Dihomo-Met	2	-	-	$C_7H_{15}NO_2S$	80	$[M+H]^+$	178.0896	178.0894	1.1	14
**10**	Hexahomo-Met *S*-Oxide	2	-	-	$C_{11}H_{23}NO_3S$	164	$[M+H]^+$	250.1471	250.1453	7.2	8.7
**11**	Pentahomo-Met *S*-Oxide	2	-	-	$C_{10}H_{21}NO_3S$	88	$[M+H]^+$	236.1315	236.1307	3.4	29
**12**	Indol-3-ylmethyl glucosinolate (I3M GLS)	2	-	[3,4]	$C_{16}H_{20}N_2O_9S_2$	167	$[M-H]^-$	447.0537	447.0529	1.8	3.4
**13**	4-Methoxy-indol-3-ylmethyl glucosinolate (4MeO-I3M GLS)	2	-	[3,4]	$C_{17}H_{22}N_2O_{10}S_2$	210	$[M-H]^-$	477.0643	477.0625	3.8	3.4
**14**	1-Methoxy-indol-3-ylmethyl glucosinolate (1MeO-I3M GLS)	2	-	[3,4]	$C_{17}H_{22}N_2O_{10}S_2$	249	$[M-H]^-$	477.0643	477.0629	2.9	12
**15**	Phenylethyl glucosinolate	2	-	[3,4]	$C_{15}H_{21}NO_9S_2$	194	$[M-H]^-$	422.0585	422.0574	2.6	9.5
**16**	3-Methylsulfinylpropyl glucosinolate (3MeSO-Propyl GLS)	2	-	[3,4]	$C_{11}H_{21}NO_{10}S_3$	40	$[M-H]^-$	422.0255	422.0247	1.9	10
**17**	4-Methylsulfinylbutyl glucosinolate (4MeSO-Butyl GLS)	2	-	[3,4]	$C_{12}H_{23}NO_{10}S_3$	40	$[M-H]^-$	436.0411	436.0432	4.8	3.9
**18**	5-Methylsulfinylpentyl glucosinolate (5MeSO-Pentyl GLS)	2	-	[3,4]	$C_{13}H_{25}NO_{10}S_3$	45	$[M-H]^-$	450.0568	450.0567	0.2	10
**19**	7-Methylsulfinylheptyl glucosinolate (7MeSO-Heptyl GLS)	2	-	[3,4]	$C_{15}H_{29}NO_{10}S_3$	146	$[M-H]^-$	478.0881	478.0866	3.1	5.0
**20**	8-Methylsulfinyloctyl glucosinolate (8MeSO-Octyl GLS)	2	-	[3,4]	$C_{16}H_{31}NO_{10}S_3$	188	$[M-H]^-$	492.1037	492.1026	2.2	2.8
**21**	4-Methylthiobutyl glucosinolate (4MeS-Butyl GLS)	2	-	[3,4]	$C_{12}H_{23}NO_9S_3$	140	$[M-H]^-$	420.0462	420.0465	0.7	10
**22**	5-Methylthiopentyl glucosinolate (5MeS-Pentyl GLS)	2	-	[3,4]	$C_{13}H_{25}NO_9S_3$	196	$[M-H]^-$	434.0619	434.0605	3.2	2.6
**23**	6-Methylthiohexyl glucosinolate (6MeS-Hexyl GLS)	2	-	[3,4]	$C_{14}H_{27}NO_9S_3$	246	$[M-H]^-$	448.0775	448.0760	3.3	15
**24**	7-Methylthioheptyl glucosinolate (7MeS-Heptyl GLS)	2	-	[3,4]	$C_{15}H_{29}NO_9S_3$	292	$[M-H]^-$	462.0932	462.0938	1.3	4.8
**25**	8-Methylthiooctyl glucosinolate (8MeS-Octyl GLS)	2	-	[3,4]	$C_{16}H_{31}NO_9S_3$	338	$[M-H]^-$	476.1088	476.1085	0.6	4.7
**26**	Glutathione-indole-3-acetonitrile conjugate (GS-IAN)	1	synthetic	[5-7]	$C_{20}H_{23}N_5O_6S$	266	$[M+H]^+$	462.1442	462.1412	6.5	4.2
**27**	Cysteine-indole-3-acetonitrile conjugate (Cys(IAN))	1	synthetic	[7]	$C_{13}H_{13}N_3O_2S$	234/240	$[M+H]^+$	276.0801	276.0790	4.0	12
**28**	*N*-Malonyl-Cys(IAN)	2	-	[7]	$C_{16}H_{15}N_3O_5S$	329	$[M+Na]^+$	384.0625	384.0605	5.2	12
**29**	3-Mercaptolactic acid-IAN conjugate (Mla(IAN))	2	-	[7]	$C_{13}H_{12}N_2O_3S$	348	$[M-H]^-$	275.0496	275.0472	8.7	9.7
**30**	Dihydrocamalexic acid (DHCA)	1	synthetic	[1,7,8]	$C_{12}H_{10}N_2O_2S$	230	$[M+H]^+$	247.0536	247.0531	2.0	5.2
**31**	DHCA-Gly conjugate	2	-	[7]	$C_{14}H_{13}N_3O_3S$	255	$[M+H]^+$	304.0750	304.0747	1.0	10
**32**	DHCA-Gln conjugate	2	-	[7]	$C_{17}H_{18}N_4O_4S$	234	$[M+H]^+$	375.1122	375.1115	1.9	14
**33**	Camalexin	1	synthetic	[7,8]	$C_{11}H_8N_2S$	398	$[M+H]^+$	201.0481	201.0477	2.0	2.3
**34**	Hydroxycamalexin-#1 (HC-#1)	2	-	-	$C_{11}H_8N_2OS$	277	$[M+H]^+$	217.0430	217.0415	6.9	6.3
**35**	HC-#1 *O*-Hexoside	2	-	[7]	$C_{17}H_{18}N_2O_6S$	226	$[M+H]^+$	379.0958	379.0948	2.6	5.3
**36**	HC-#1 *O*-Malonylhexoside	2	-	[7]	$C_{20}H_{20}N_2O_9S$	270	$[M+H]^+$	465.0962	465.0945	3.7	5.3
**37**	HC-#1 *O*-Malylhexoside	2	-	-	$C_{21}H_{22}N_2O_{10}S$	174	$[M+H]^+$	495.1068	495.1050	3.6	23

**38**	Hydroxycamalexin-#2 (HC-#2)	2	-	-	$C_{11}H_8N_2OS$	323	$[M+H]^+$	217.0430	217.0423	3.2	6.1
**39**	HC-#2 *O*-Hexoside	2	-	[7]	$C_{17}H_{18}N_2O_6S$	281	$[M+H]^+$	379.0958	379..0946	3.2	2.0
**40**	HC-#2 *O*-Malonylhexoside	2	-	[7]	$C_{20}H_{20}N_2O_9S$	313	$[M+H]^+$	465.0962	465.0948	3.0	3.6
**41**	HC-#2 *O*-Bis(malonyl)hexoside	2	-	-	$C_{23}H_{22}N_2O_{12}S$	340	$[M+H]^+$	551.0966	551.0953	2.4	7.3
**42**	HC-#2 *O*-Malylhexoside (HC-#2 Malyl-Hex)	2	-	-	$C_{21}H_{22}N_2O_{10}S$	220	$[M+H]^+$	495.1068	495.1068	0.0	27
**43**	HC-#2 *O*-(Malyl-malonylhexoside)	2	-	-	$C_{24}H_{24}N_2O_{13}S$	256	$[M+H]^+$	581.1072	581.1047	4.3	7.7
**44**	Indole-3-carboxylic acid (I3COOH)	1	Sigma-Aldrich	[1,9,10]	$C_9H_7NO_2$	284	$[M+H]^+$	162.0550	162.0541	5.6	18
**45**	Methyl indole-3-carboxylate (I3COOMe)	1	OlChemIm	[7,10]	$C_{10}H_9NO_2$	400	$[M+H]^+$	176.0706	176.0695	6.2	5.2
**46**	β-Glucosyl indole-3-carboxylate (I3COOGlc)	2	-	[1,7,9,10]	$C_{15}H_{17}NO_7$	225	$[M-H]^-$	322.0932	322.0928	1.2	8.8
**47**	6-β-Glucosyloxy-indole-3-carboxylic acid (6GlcO-I3COOH)	2	-	[1,7,9,10]	$C_{15}H_{17}NO_8$	100	$[M-H]^-$	338.0881	338.0872	2.7	2.0
**48**	β-Glucosyl 6-hydroxy-indole-3-carboxylate (6HO-I3COOGlc)	2	-	[1,7,9,10]	$C_{15}H_{17}NO_8$	114	$[M-H]^-$	338.0881	338.0865	4.7	11
**49**	β-Glucosyl 6-β-Glucosyloxy-indole-3-carboxylate (6GlcO-I3COOGlc)	2	-	[10]	$C_{21}H_{27}NO_{13}$	62	$[M-H]^-$	500.1410	500.1400	2.0	16
**50**	1-Methyl-indole-3-carboxylic acid (1Me-I3COOH)	1	Sigma-Aldrich	[1]	$C_{10}H_9NO_2$	355	$[M+H]^+$	176.0706	176.0704	1.1	7.5
**51**	Hexosyl 1-methyl-indole-3-carboxylate (1Me-I3COOHex)	2	-	-	$C_{16}H_{19}NO_7$	274	$[M-H+FA]^-$	382.1144	382.1121	6.0	2.9
**52**	Indol-3-ylmethyl amine ($I3MNH_2$)	1	Sigma-Aldrich	[2]	$C_9H_{10}N_2$	114	$[M+H-NH_3]^+$	130.0651	130.0638	10.0	17
**53**	4-Methoxy-indol-3-ylmethyl amine ($4MeO\text{-}I3MNH_2$)	1	synthetic	[2]	$C_{10}H_{12}N_2O$	193	$[M+H-NH_3]^+$	160.0757	160.0748	5.6	8.1
**54**	4-Methoxy-indole-3-carbaldehyde (4MeO-I3CHO)	1	Chem-Impex	[1,10]	$C_{10}H_9NO_2$	334	$[M+H]^+$	176.0706	176.0689	9.7	7.1
**55**	Dihydroascorbigen *O*-Hexoside (DihydroAsc Hex)	2	-	[10]	$C_{21}H_{27}NO_{11}$	206	$[M-H]^-$	468.1511	468.1496	3.2	4.7
**56**	7-Methylsulfinyloctyl amine ($7MeSO\text{-}Heptyl\text{-}NH_2$)	1	synthetic	[11]	$C_8H_{19}NOS$	60	$[M+H]^+$	178.1260	178.1256	2.2	16
**57**	8-Methylsulfinyloctyl amine ($8MeSO\text{-}Octyl\text{-}NH_2$)	1	synthetic	[11]	$C_9H_{21}NOS$	136	$[M+H]^+$	192.1417	192.1408	4.7	5.0
**58**	Acetylagmatine	2	-	-	$C_7H_{16}N_4O$	40	$[M+H]^+$	173.1397	173.1402	2.9	5.5
**59**	Phenacetylagmatine	2	-	-	$C_{13}H_{20}N_4O$	202	$[M+H]^+$	249.1710	249.1702	3.2	5.5
**60**	Coumaroylagmatine	2	-	[12]	$C_{14}H_{20}N_4O_2$	172/201	$[M+H]^+$	277.1659	277.1649	3.6	10
**61**	Feruloylagmatine	2	-	[12]	$C_{15}H_{22}N_4O_3$	190/215	$[M+H]^+$	307.1765	307.1753	3.9	23
**62**	*N*-Phenacetyl-Asp	2	-	-	$C_{12}H_{13}NO_5$	216	$[M-H]^-$	250.0721	250.0710	4.4	36
**63**	Salicylic Acid (SA)	1	Sigma-Aldrich	-	$C_7H_6O_3$	298	$[M-H]^-$	137.0244	137.0229	10.9	11
**64**	1-*O*-Salicoyl-β-glucose (SGE)	2	-	[14]	$C_{13}H_{16}O_8$	216	$[M-H]^-$	299.0772	299.0772	0.0	interf.
**65**	Salicylic acid 2-*O*-β-glucoside (SAG)	2	-	[14]	$C_{13}H_{16}O_8$	170	$[M-H]^-$	299.0772	299.0758	4.7	4.6
**66**	2,5-Dihydroxybenzoic acid *O*-Hexoside (2,5-DHBA Hex)	2	-	[14]	$C_{13}H_{16}O_9$	87	$[M-H]^-$	315.0722	315.0700	7.0	20
**67**	2,5-Dihydroxybenzoic acid *O*-Pentoside (2,5-DHBA Pent)	2	-	[14]	$C_{12}H_{14}O_8$	150	$[M-H]^-$	285.0616	285.0605	3.9	1.7
**68**	2,3-Dihydroxybenzoic acid 3-*O*-β-Xyloside (2,3-DHBA 3-Xyl)	1	isolated	[13,14]	$C_{12}H_{14}O_8$	176	$[M-H]^-$	285.0616	285.0604	4.2	19
**69**	9,12,13-Trihydroxy-10,15-octadecadienoic acid	2	-	[15]	$C_{18}H_{32}O_5$	415/420	$[M-H]^-$	327.2177	327.2154	7.0	5.7
**70**	Oxo-phytodienoic acid (oPDA)	2	-	[16]	$C_{18}H_{28}O_3$	611	$[M-H]^-$	291.1966	291.1957	3.1	2.2
**71**	Jasmonic acid (JA)	1	Sigma-Aldrich	[16]	$C_{12}H_{18}O_3$	402	$[M-H]^-$	209.1183	209.1178	2.4	36
**72**	1-*O*-Jasmonyl-β-glucose (JA Glc)	2	-	[16]	$C_{18}H_{28}O_8$	320	$[M-H]^-$	371.1711	371.1671	10.8	interf.
**73**	Feruloylmalate (FerM)	2	-	-	$C_{14}H_{14}O_8$	275	$[M-H]^-$	309.0616	309.0633	5.5	14
**74**	Coniferyl alcohol(8-*O*-4)feruloylmalate (G(8-*O*-4)FerM)	2	-	[17]	$C_{24}H_{26}O_{12}$	280/292	$[M-H]^-$	505.1351	505.1338	2.6	13
**75**	Sinapyl alcohol(8-*O*-4)feruloylmalate (S(8-*O*-4)FerM)	2	-	-	$C_{25}H_{28}O_{13}$	273/278	$[M-H]^-$	535.1457	535.1433	4.5	36
**76**	Coniferyl alcohol(8-5)feruloylmalate (G(8-5)FerM)	2	-	[17]	$C_{24}H_{24}O_{11}$	349	$[M-H]^-$	487.1246	487.1246	0.0	11
**77**	5-Hydroxyferuloylmalate (5HO-FerM)	2	-	-	$C_{14}H_{14}O_9$	234	$[M-H]^-$	325.0565	325.0555	3.1	6.0

**78**	Sinapoyl malate (SinM)	2	-	-	$C_{15}H_{16}O_9$	278	$[M-H]^-$	339.0722	339.0710	3.5	4.4
**79**	Coniferyl alcohol(8-*O*-4)sinapoylmalate (G(8-*O*-4)SinM)	2	-	-	$C_{25}H_{28}O_{13}$	309/324	$[M-H]^-$	535.1457	535.1454	0.6	16
**80**	1-*O*-Sinapoyl-β-glucose (SinGlc)	1	isolated	[18]	$C_{17}H_{22}O_{10}$	216	$[M-H]^-$	385.1140	385.1139	0.3	19
**81**	1,2-Di-*O*-sinapoyl-β-glucose (DiSinGlc)	1	isolated	[18,19]	$C_{28}H_{32}O_{14}$	318	$[M-H]^-$	591.1719	591.1706	2.2	7.1
**82**	Kaempferol 3,7-di-*O*-α-Rha (K 3-Rha-7-Rha)	2	-	[20]	$C_{27}H_{30}O_{14}$	283	$[M-H]^-$	577.1563	577.1563	0.0	4.7
**83**	Kaempferol 3-*O*-β-Glc-7-*O*-α-Rha (K 3-Glc-7-Rha)	2	-	[20]	$C_{27}H_{30}O_{15}$	259	$[M-H]^-$	593.1512	593.1510	0.3	6.4
**84**	Quercetin 3-*O*-β-Glc-7-*O*-α-Rha (Q 3-Glc-7-Rha)	2	-	[20]	$C_{27}H_{30}O_{16}$	242	$[M-H]^-$	609.1461	609.1442	3.1	8.9
**85**	Kaempferol 3-*O*-β-(α-Rha(1→2)-Glc)-7-*O*-α-Rha (K 3-(Rha-Glc)-7-Rha)	2	-	[20]	$C_{33}H_{40}O_{19}$	227	$[M-H]^-$	739.2091	739.2090	0.1	3.3
**86**	Quercetin 3-*O*-(Deoxyhex-Hex)-7-*O*-Deoxyhex (Q 3-(DeoxyHex-Hex)-7-DeoxyHex)	2	-	-	$C_{33}H_{40}O_{20}$	216	$[M-H]^-$	755.2040	755.2023	2.3	13
**87**	Kaempferol 3-*O*-β-(β-Glc(1→6)-Glc)-7-*O*-α-Rha (K 3-(Glc-Glc)-7-Rha)	2	-	[21]	$C_{33}H_{40}O_{20}$	251	$[M-H]^-$	755.2040	755.2015	3.3	22
**88**	nonfluorescent chlorophyll catabolite 1 (At-NCC-1)	2	-	[22]	$C_{40}H_{48}N_4O_{13}$	339	$[M-H]^-$	791.3145	791.3112	4.2	8.8
**89**	nonfluorescent chlorophyll catabolite 4 (At-NCC-4)	2	-	[22]	$C_{41}H_{50}N_4O_{13}$	366	$[M-H]^-$	805.3302	805.3241	7.6	9.9
**90**[e]	UNK-1 (indolic)	3	-	[7]	$C_{11}H_{10}N_2O$	222	$[M+H]^+$	187.0866	187.0856	5.3	2.1
**91**	UNK-2 (indolic)	3	-	[10]	$C_{12}H_{12}N_2O_4$	180	$[M+H]^+$	249.0870	249.0856	5.6	4.4
**92**	UNK-3 (Hexoside, indolic aglycone $C_9H_8N_2O_2$)	3	-	-	$C_{15}H_{18}N_2O_7$	166	$[M-H]^-$	337.1041	337.1024	5.0	24
**93**	UNK-4 (Hexoside, indolic aglycone $C_9H_7NO_3$)	3	-	[10]	$C_{15}H_{17}NO_8$	175	$[M-H]^-$	338.0881	338.0866	4.4	32
**94**	UNK-5 (Hexoside, indolic aglycone $C_{10}H_9NO_3$)	3	-	-	$C_{16}H_{19}NO_8$	224	$[M-H]^-$	352.1038	352.1026	3.4	18
**95**	UNK-6	4	-	-	$C_{10}H_9NO_3$	406	$[M+H]^+$	192.0655	192.0652	1.6	7.6
**96**	UNK-7	4	-	-	$C_{10}H_9NO_3$	366	$[M+H]^+$	192.0655	192.0648	3.6	6.7
**97**	UNK-8	4	-	-	$C_{11}H_{11}NO_3$	486	$[M+H]^+$	206.0812	206.0803	4.4	11
**98**	UNK-9 (Hydroxycamalexin-#3)	3	-	-	$C_{11}H_8N_2OS$	441	$[M+H]^+$	217.0430	217.0425	2.3	3.8
**99**	UNK-10 (Hexoside, aglycone $C_6H_{11}NO_3$)	3	-	-	$C_{12}H_{21}NO_8$	94	$[M+H]^+$	308.1340	308.1334	1.9	8.7
**100**	UNK-11 (Malate ester)	3	-	-	$C_{16}H_{20}O_8$	365/380	$[M-H]^-$	339.1085	339.1089	1.2	7.0
**101**	UNK-12	4	-	-	$C_{10}H_{20}N_2O_3S$	159	$[M+H]^+$	249.1267	249.1263	1.6	13
**102**	UNK-13	4	-	-	$C_{13}H_{10}N_2O_4S$	305	$[M+H]^+$	291.0434	291.0425	3.1	14
**103**	UNK-14	4	-	-	$C_{12}H_{19}N_3O_5$	60	$[M-H]^-$	284.1252	284.1244	2.8	5.2
**104**	UNK-15	4	-	-	$C_{17}H_{17}N_3O_5S_2$	273	$[M+H]^+$	408.0682	408.0671	2.7	6.4
**105**	UNK-16	4	-	-	$C_{15}H_{13}N_3O_5S$	295	$[M-H]^-$	346.0503	346.0487	4.6	18
**106**	UNK-17	4	-	-	$C_{15}H_{16}N_2O_6$	158	$[M-H]^-$	319.0936	319.0929	2.2	9.3
**107**	UNK-18	4	-	-	$C_{12}H_{20}N_2O_4$	201	$[M-H]^-$	255.1350	255.1352	0.8	4.2
**108**	UNK-19	4	-	-	$C_{17}H_{22}N_2O_7$	201	$[M-H]^-$	365.1354	365.1330	6.6	17
**109**	UNK-20 ($C_{11}H_{20}O_3$, sulfated)	4	-	-	$C_{11}H_{20}O_6S$	320	$[M-H]^-$	279.0908	279.0890	6.4	10
**110**	UNK-21	4	-	-	$C_{16}H_{19}NO_9$	216	$[M-H]^-$	368.0987	368.0977	2.7	9.5
**111**	UNK-22 (Dihexoside, aglycone $C_9H_7NO_2$)	3	-	-	$C_{21}H_{27}NO_{12}$	192/197	$[M-H]^-$	484.1460	484.1435	5.2	16
**112**	UNK-23 (Dihexoside, aglycone $C_9H_7NO_2$)	3	-	-	$C_{21}H_{27}NO_{12}$	230/235	$[M-H]^-$	484.1460	484.1438	4.5	16
**113**	UNK-24 (Dihexoside, aglycone $C_9H_7NO_3$)	3	-	-	$C_{21}H_{27}NO_{13}$	242/247	$[M-H]^-$	500.1410	500.1386	4.8	12

[a]Annotation level according to Sumner et al. *Metabolomics* **2007**, *3*, 211: **1**, identified compound; **2**, putatively annotated compound, **3**, putatively characterised compound class, **4**, unknown compound.

[b]Literature: [1] Bednarek et al. *Plant Physiol.* **2005**, *138*, 1058; [2] Bednarek et al. *Science* **2009**, *323*, 101; [3] Petersen et al. *Planta* 214, 562; [4] Brown et al. *Phytochemistry* **2003,** *62*, 471; [5] Geu-Flores et al. *Plant Cell* **2011**, *23*, 2456; [6] Su et al. *Plant Cell* **2011**, *23*, 364; [7] Böttcher et al. *Plant Cell* **2009**, *21*, 1830; [8] Schuhegger et al. *Plant Physiol.* **2006**, *141*, 1248; [9] Hagemeier et al. *PNAS* **2001**, *98*, 753; [10] Böttcher er al. *Plant Phys.* **2014**, *165*, 841; [11] prepared by acidic hydrolysis of 7-methylsulfinylheptyl isothiocyanate/8-methylsulfinyloctyl isothiocyanate; [12] Muroi et al. *Planta* **2009**, *230*, 517; [13] Zhang et al. *PNAS* **2013**, *110*, 14807;

[14] Bartsch et al. *J. Biol.Chem.* **2010**, *285*, 25654; [15] Floerl et al. *PloS One* **2012**, *7*, e31435; [16] Göbel et al. *Phytochemistry*, **2009**, *70*, 1485 and references cited therein; [17] Rhode et al. *Plant Cell* **2004**, *16*, 2749; [18] isolated from *Brassica napus* seeds, Baumert et al., *Phytochemistry* **2005**, *66*, 1334; [19] Fraser et al. *Plant Physiol.* **2007**, *144*, 1986; [20] Yonekura-Sakakibara et al. *Plant Cell* **2008**, *20*, 2160; [21] Veit et al. *J. Nat. Prod.* **1999**, *62*, 1301; [22] Pružinská et al. *Plant Physiol.* **2005**, *139*, 52

[c] Absolute mass deviation in ppm

[d] Goodness of fit between measured and calculated isotope pattern (Bruker Daltonics, DataAnalysis 4.0); interf. = mSigma could not be determined due to the presence of interfering ion species

[e] UNK = unknown compound or only putatively characterised (see annotation level [a] in column 2)

SUPPLEMENTAL TABLE 2. Collision-induced dissociation (CID) mass spectral data of annotated compounds.

no.	compound name	elemental composition	precursor CE [eV]	observed fragment ions upon CID[a] *m/z*(rel. int. [%], elemental composition), precursor ion marked in bold
**1**	Phe	$C_9H_{11}NO_2$	$[M+H]^+$, 10	identical CID mass spectrum as observed for authenticated standard
**2**	*N*-Malonyl-Phe	$C_{12}H_{13}NO_5$	$[M+H]^+$, 15	**252** (0, $C_{12}H_{14}NO_5^+$), 206 (75, $C_{11}H_{12}NO_3^+$), 188 (62, $C_{11}H_{10}NO_2^+$), 166 (5, $C_9H_{12}NO_2^+$), 146 (35, $C_9H_8NO^+$), 120 (100, $C_8H_{10}N^+$)
			$[M-H]^-$, 10	**250** (0, $C_{12}H_{12}NO_5^-$), 206 (100, $C_{11}H_{12}NO_3^-$), 164 (47, $C_9H_{10}NO_2^-$), 147 (8, $C_9H_7O_2^-$)
**3**	γ-Glu-Phe	$C_{14}H_{18}N_2O_5$	$[M+H]^+$, 15	**295** (5, $C_{14}H_{19}N_2O_5^+$), 278 (25, $C_{14}H_{16}NO_5^+$), 232 (22, $C_{13}H_{14}NO_3^+$), 186 (16, $C_{12}H_{12}NO^+$), 166 (87, $C_9H_{12}NO_2^+$), 149 (8, $C_9H_9NO_2^+$), 131 (6, $C_9H_7O^+$), 130 (12, $C_5H_8NO_3^+$), 120 (100, $C_8H_{10}N^+$)
**4**	Tyr	$C_9H_{11}NO_3$	$[M+H]^+$, 10	identical CID mass spectrum as observed for authenticated standard
**5**	Trp	$C_{11}H_{12}N_2O_2$	$[M+H]^+$, 10	identical CID mass spectrum as observed for authenticated standard
**6**	Leu/Ile	$C_6H_{13}NO_2$	$[M+H]^+$, 10	identical CID mass spectrum as observed for authenticated standard
**7**	Glutathione (GS-H)	$C_{10}H_{17}N_3O_6S$	$[M+H]^+$, 15	identical CID mass spectrum as observed for authenticated standard
**8**	Raphanusamic Acid	$C_4H_5NO_2S_2$	$[M+H]^+$, 10	**164** (41, $C_4H_6NO_2S_2^+$), 118 (100, $C_3H_4NS_2^+$), 105 (4, $C_3H_5O_2S^+$)
**9**	Dihomo-Met	$C_7H_{15}NO_2S$	$[M+H]^+$, 10	**178** (19, $C_7H_{16}NO_2S^+$), 161 (100, $C_7H_{13}O_2S^+$), 132 (56, $C_6H_{14}NS^+$), 130 (16, $C_6H_{12}NO_2^+$), 115 (11, $C_6H_{11}S^+$), 84 (36, $C_5H_{10}N^+$)
**10**	Hexahomo-Met *S*-Oxide	$C_{11}H_{23}NO_3S$	$[M+H]^+$, 15	**250** (3, $C_{11}H_{24}NO_3S^+$), 204 (100, $C_{10}H_{22}NOS^+$), 186 (4, $C_{10}H_{20}NS^+$), 140 (15, $C_9H_{18}N^+$)
**11**	Pentahomo-Met *S*-Oxide	$C_{10}H_{21}NO_3S$	$[M+H]^+$, 15	**236** (2, $C_{10}H_{22}NO_3S^+$), 190 (100, $C_9H_{20}NOS^+$), 172 (4, $C_9H_{18}NS^+$), 126 (10, $C_8H_{16}N^+$)
**12**	I3M GLS	$C_{16}H_{20}N_2O_9S_2$	$[M-H]^-$, 25	characteristic ions at *m/z* 259 ($C_6H_{11}O_9S^-$) and *m/z* 97 (HSO_4^-) were observed, see reference [1]
**13**	4MeO-I3M GLS	$C_{17}H_{22}N_2O_{10}S_2$	$[M-H]^-$, 25	characteristic ions at *m/z* 259 ($C_6H_{11}O_9S^-$) and *m/z* 97 (HSO_4^-) were observed, see reference [1]
**14**	1MeO-I3M GLS	$C_{17}H_{22}N_2O_{10}S_2$	$[M-H]^-$, 25	characteristic ions at *m/z* 259 ($C_6H_{11}O_9S^-$) and *m/z* 97 (HSO_4^-) were observed, see reference [1]
**15**	Phenylethyl glucosinolate	$C_{15}H_{21}NO_9S_2$	$[M-H]^-$, 25	characteristic ions at *m/z* 259 ($C_6H_{11}O_9S^-$) and *m/z* 97 (HSO_4^-) were observed, see reference [1]
**16**	3MeSO-Propyl GLS	$C_{11}H_{21}NO_{10}S_3$	$[M-H]^-$, 25	characteristic ions at *m/z* 259 ($C_6H_{11}O_9S^-$) and *m/z* 97 (HSO_4^-) were observed, see reference [1]
**17**	4MeSO-Butyl GLS	$C_{12}H_{23}NO_{10}S_3$	$[M-H]^-$, 25	characteristic ions at *m/z* 259 ($C_6H_{11}O_9S^-$) and *m/z* 97 (HSO_4^-) were observed, see reference [1]
**18**	5MeSO-Pentyl GLS	$C_{13}H_{25}NO_{10}S_3$	$[M-H]^-$, 25	characteristic ions at *m/z* 259 ($C_6H_{11}O_9S^-$) and *m/z* 97 (HSO_4^-) were observed, see reference [1]
**19**	7MeSO-Heptyl GLS	$C_{15}H_{29}NO_{10}S_3$	$[M-H]^-$, 25	characteristic ions at *m/z* 259 ($C_6H_{11}O_9S^-$) and *m/z* 97 (HSO_4^-) were observed, see reference [1]
**20**	8MeSO-Octyl GLS	$C_{16}H_{31}NO_{10}S_3$	$[M-H]^-$, 25	characteristic ions at *m/z* 259 ($C_6H_{11}O_9S^-$) and *m/z* 97 (HSO_4^-) were observed, see reference [1]
**21**	4MeS-Butyl GLS	$C_{12}H_{23}NO_9S_3$	$[M-H]^-$, 25	characteristic ions at *m/z* 259 ($C_6H_{11}O_9S^-$) and *m/z* 97 (HSO_4^-) were observed, see reference [1]
**22**	5MeS-Pentyl GLS	$C_{13}H_{25}NO_9S_3$	$[M-H]^-$, 25	characteristic ions at *m/z* 259 ($C_6H_{11}O_9S^-$) and *m/z* 97 (HSO_4^-) were observed, see reference [1]
**23**	6MeS-Hexyl GLS	$C_{14}H_{27}NO_9S_3$	$[M-H]^-$, 25	characteristic ions at *m/z* 259 ($C_6H_{11}O_9S^-$) and *m/z* 97 (HSO_4^-) were observed, see reference [1]
**24**	7MeS-Heptyl GLS	$C_{15}H_{29}NO_9S_3$	$[M-H]^-$, 25	characteristic ions at *m/z* 259 ($C_6H_{11}O_9S^-$) and *m/z* 97 (HSO_4^-) were observed, see reference [1]
**25**	8MeS-Octyl GLS	$C_{16}H_{31}NO_9S_3$	$[M-H]^-$, 25	characteristic ions at *m/z* 259 ($C_6H_{11}O_9S^-$) and *m/z* 97 (HSO_4^-) were observed, see reference [1]
**26**	GS-IAN	$C_{20}H_{23}N_5O_6S$	$[M+H]^+$, 15	identical CID mass spectrum as observed for authenticated standard, see reference [2]
**27**	Cys(IAN)	$C_{13}H_{13}N_3O_2S$	$[M+H]^+$, 20	identical CID mass spectrum as observed for authenticated standard, see reference [2]
**28**	*N*-Malonyl-Cys(IAN)	$C_{16}H_{15}N_3O_5S$	$[M-H]^-$, 10	identical CID mass spectrum as previously reported, see reference [2]
**29**	Mla(IAN)	$C_{13}H_{12}N_2O_3S$	$[M-H]^-$, 15	identical CID mass spectrum as previously reported, see reference [2]
**30**	DHCA	$C_{12}H_{10}N_2O_2S$	$[M+H]^+$, 15	identical CID mass spectrum as observed for authenticated standard, see reference [2]
**31**	DHCA-Gly conjugate	$C_{14}H_{13}N_3O_3S$	$[M+H]^+$, 20	**304** (19, $C_{14}H_{14}N_3O_3S^+$), 258 (3, $C_{13}H_{12}N_3OS^+$), 201 (100, $C_{11}H_9N_2S^+$), 162 (20, $C_5H_8NO_3S^+$), 144 (16, $C_5H_6NO_2S^+$), 143 (35, $C_9H_7N_2^+$), 116 (32, $C_4H_6NOS^+$)
**32**	DHCA-Gln conjugate	$C_{17}H_{18}N_4O_4S$	$[M+H]^+$, 20	identical CID mass spectrum as previously reported, see reference [2]
**33**	Camalexin	$C_{11}H_8N_2S$	$[M+H]^+$, 20	identical CID mass spectrum as observed for authenticated standard, see reference [2]
**34**	Hydroxycamalexin-#1 (HC-#1)	$C_{11}H_8N_2OS$	$[M+H]^+$, 25	**217** (100, $C_{11}H_9N_2OS^+$), 216 (21, $C_{11}H_8N_2OS^{+\bullet}$), 200 (16, $C_{11}H_8N_2S^{+\bullet}$), 199 (9, $C_{11}H_7N_2S^+$), 198 (4, $C_{11}H_7N_2S^{+\bullet}$), 190 (20, $C_{10}H_8NOS^+$), 189 (26, $C_{10}H_9N_2S^+$), 184 (10, $C_{11}H_8N_2O^{+\bullet}$), 176 (21, $C_9H_6NOS^+$), 173 (8, $C_{10}H_7NS^{+\bullet}$), 172 (6, $C_{10}H_6NS^+$), 162 (11, $C_9H_8NS^+$), 158 (67, $C_9H_6N_2O^{+\bullet}$), 156 (14, $C_{10}H_8N_2^{+\bullet}$), 146 (5, $C_9H_8NO^+$), 133 (17, $C_8H_7NO^{+\bullet}$), 132 (21, $C_8H_6NO^+$), 104 (7, $C_7H_6N^+$), 98 (2, $C_4H_4NS^+$), 59 (4, $C_2H_3S^+$)
**35**	HC-#1 *O*-Hexoside	$C_{17}H_{18}N_2O_6S$	$[M+H]^+$, 10	**379** (100, $C_{17}H_{19}N_2O_6S^+$), 217 (56, $C_{11}H_9N_2OS^+$), see reference [2]
**36**	HC-#1 *O*-Malonylhexoside	$C_{20}H_{20}N_2O_9S$	$[M+H]^+$, 15	**465** (100, $C_{20}H_{21}N_2O_9S^+$), 421 (19, $C_{19}H_{21}N_2O_7S^+$), 379 (1, $C_{17}H_{19}N_2O_6S^+$), 217 (75, $C_{11}H_9N_2OS^+$)

**37**	HC-#1 *O*-Malylhexoside	$C_{21}H_{22}N_2O_{10}S$	$[M+H]^+$, 10	**495** (100, $C_{21}H_{23}N_2O_{10}S^+$), 451 (21, $C_{20}H_{23}N_2O_8S^+$), 379 (49, $C_{17}H_{19}N_2O_6S^+$), 333 (47, $C_{15}H_{13}N_2O_5S^+$), 289 (3, $C_{14}H_{13}N_2O_3S^+$), 245 (13, $C_{13}H_{13}N_2OS^+$), 217 (8, $C_{11}H_9N_2OS^+$)
**38**	Hydroxycamalexin-#2 (HC-#2)	$C_{11}H_8N_2OS$	$[M+H]^+$, 25	**217** (100, $C_{11}H_9N_2OS^+$), 199 (37, $C_{11}H_7N_2S^+$), 198 (23, $C_{11}H_7N_2S^{+\bullet}$), 189 (97, $C_{10}H_9N_2S^+$), 184 (10, $C_{11}H_8N_2O^{+\bullet}$), 176 (6, $C_9H_6NOS^+$), 173 (18, $C_{10}H_7NS^{+\bullet}$), 172 (20, $C_{10}H_6NS^+$), 162 (41, $C_9H_8NS^+$), 158 (40, $C_9H_6N_2O^{+\bullet}$), 156 (64, $C_{10}H_8N_2^{+\bullet}$), 148 (5, $C_8H_6NS^+$), 132 (11, $C_8H_6NO^+$), 118 (4, $C_8H_8N^+$), 117 (5, $C_8H_7N^{+\bullet}$), 104 (24, $C_7H_6N^+$), 98 (3, $C_4H_4NS^+$), 59 (4, $C_2H_3S^+$)
**39**	HC-#2 *O*-Hexoside	$C_{17}H_{18}N_2O_6S$	$[M+H]^+$, 10	**379** (100, $C_{17}H_{19}N_2O_6S^+$), 217 (25, $C_{11}H_9N_2OS^+$), see reference [2]
**40**	HC-#2 *O*-Malonylhexoside	$C_{20}H_{20}N_2O_9S$	$[M+H]^+$, 15	**465** (100, $C_{20}H_{21}N_2O_9S^+$), 421 (36, $C_{19}H_{21}N_2O_7S^+$), 379 (4, $C_{17}H_{19}N_2O_6S^+$), 217 (76, $C_{11}H_9N_2OS^+$), see reference [2]
**41**	HC-#2 *O*-Bis(malonyl)hexoside	$C_{23}H_{22}N_2O_{12}S$	$[M+H]^+$, 20	**551** (51, $C_{23}H_{23}N_2O_{12}S^+$), 507 (18, $C_{22}H_{23}N_2O_{10}S^+$), 465 (3, $C_{20}H_{21}N_2O_9S^+$), 463 (2, $C_{21}H_{23}N_2O_8S^+$), 317 (1, $C_{12}H_{13}O_{10}^+$), 273 (1, $C_{11}H_{13}O_8^+$), 231 (2, $C_9H_{11}O_7^+$), 217 (100, $C_{11}H_9N_2OS^+$), 213 (1, $C_9H_9O_6^+$), 187 (1, $C_8H_{11}O_5^+$), 127 (2, $C_6H_7O_3^+$)
**42**	HC-#2 *O*-Malylhexoside	$C_{21}H_{22}N_2O_{10}S$	$[M+H]^+$, 10	**495** (26, $C_{21}H_{23}N_2O_{10}S^+$), 447 (5, $C_{21}H_{21}N_2O_9S^+$), 451 (56, $C_{20}H_{23}N_2O_8S^+$), 407 (5, $C_{19}H_{23}N_2O_6S^+$), 379 (100, $C_{17}H_{19}N_2O_6S^+$), 333 (47, $C_{15}H_{13}N_2O_5S^+$), 315 (4, $C_{15}H_{11}N_2O_4S^+$), 289 (11, $C_{14}H_{13}N_2O_3S^+$), 245 (8, $C_{13}H_{13}N_2OS^+$), 217 (10, $C_{11}H_9N_2OS^+$)
**43**	HC-#2 *O*-(Malyl-malonylhexoside)	$C_{24}H_{24}N_2O_{13}S$	$[M+H]^+$, 15	**581** (8, $C_{24}H_{25}N_2O_{13}S^+$), 563 (5, $C_{24}H_{23}N_2O_{12}S^+$), 537 (59, $C_{23}H_{25}N_2O_{11}S^+$), 493 (17, $C_{22}H_{25}N_2O_9S^+$), 465 (100, $C_{20}H_{21}N_2O_9S^+$), 421 (8, $C_{19}H_{21}N_2O_7S^+$), 315 (6, $C_{15}H_{11}N_2O_4S^+$), 289 (22, $C_{14}H_{13}N_2O_3S^+$), 245 (15, $C_{13}H_{13}N_2OS^+$), 217 (7, $C_{11}H_9N_2OS^+$)
**44**	I3COOH	$C_9H_7NO_2$	$[M+H]^+$, 10	**162** (61, $C_9H_8NO_2^+$), 144 (56, $C_9H_6NO^+$), 118 (100, $C_8H_8N^+$), 116 (9, $C_8H_6N^+$), see reference [3]
**45**	I3COOMe	$C_{10}H_9NO_2$	$[M+H]^+$, 10	**176** (41, $C_{10}H_{10}NO_2^+$), 162 (21, $C_9H_8NO_2^+$), 144 (100, $C_9H_6NO^+$), 132 (25, $C_9H_{10}N^+$), 117 (13, $C_8H_7N^{+\bullet}$), 116 (4, $C_8H_6N^+$), see reference [3]
**46**	I3COOGlc	$C_{15}H_{17}NO_7$	$[M-H]^-$, 10	**322** (100, $C_{15}H_{16}NO_7^-$), 262 (24, $C_{13}H_{12}NO_5^-$), 232 (3, $C_{12}H_{10}NO_4^-$), 202 (6, $C_{11}H_8NO_3^-$), 160 (9, $C_9H_6NO_2^-$), see reference [3]
**47**	6GlcO-I3COOH	$C_{15}H_{17}NO_8$	$[M-H]^-$, 10	**338** (100, $C_{15}H_{16}NO_8^-$), 176 (159, $C_9H_6NO_3^-$), 132.04 (2, $C_8H_6NO^-$), see reference [3]
**48**	6HO-I3COOGlc	$C_{15}H_{17}NO_8$	$[M-H]^-$, 10	**338** (100, $C_{15}H_{16}NO_8^-$), 278 (15, $C_{13}H_{12}NO_6^-$), 248 (2, $C_{12}H_{10}NO_5^-$), 218 (5, $C_{11}H_8NO_4^-$), 179 (1, $C_6H_{11}O_6^-$), 176 (10, $C_9H_6NO_3^-$), see reference [3]
**49**	6GlcO-I3COOGlc	$C_{21}H_{27}NO_{13}$	$[M-H]^-$, 20	**500** (100, $C_{21}H_{26}NO_{13}^-$), 440 (41, $C_{19}H_{22}NO_{11}^-$), 410 (5, $C_{18}H_{20}NO_{10}^-$), 380 (24, $C_{17}H_{18}NO_9^-$), 338 (80, $C_{15}H_{16}NO_8^-$), 278 (12, $C_{13}H_{12}NO_6^-$), 218 (7, $C_{11}H_8NO_4^-$), 176 (40, $C_9H_6NO_3^-$), 101.03 (4, $C_4H_5O_3^-$), see reference [3]
**50**	1Me-I3COOH	$C_{10}H_9NO_2$	$[M+H]^+$, 10	**176** (40, $C_{10}H_{10}NO_2^+$), 158 (12, $C_{10}H_8NO^+$), 132 (100, $C_9H_{10}N^+$), 117 (9, $C_8H_7N^{+\bullet}$)
**51**	1Me-I3COOHex	$C_{16}H_{19}NO_7$	$[M-H+FA]^-$, 10	**382** (100, $C_{17}H_{20}NO_9^-$), 336 (34, $C_{16}H_{18}NO_7^-$), 207 (35, $C_7H_{11}O_7^-$), 174 (50, $C_{10}H_8NO_2^-$)
**52**	$I3MNH_2$	$C_9H_{10}N_2$	$[M+H-NH_3]^+$, 20	**130** (100, $C_9H_8N^+$), 128 (6, $C_9H_6N^+$), 103 (11, $C_8H_7^+$), 77 (2, $C_6H_5^+$)
**53**	$4MeO-I3MNH_2$	$C_{10}H_{12}N_2O$	$[M+H-NH_3]^+$, 10	**160** (100, $C_{10}H_{10}NO^+$), 148 (8, $C_9H_{10}NO^+$), 145 (4, $C_9H_7NO^{+\bullet}$), 132 (21, $C_9H_{10}N^+$), 130 (35, $C_9H_8N^+$), 117 (4, $C_8H_7N^{+\bullet}$)
**54**	4MeO-I3CHO	$C_{10}H_9NO_2$	$[M+H]^+$, 10	**176** (100, $C_{10}H_{10}NO_2^+$), 161 (35, $C_9H_7NO_2^{+\bullet}$), 148 (23, $C_9H_{10}NO^+$), 133 (6, $C_8H_7NO^{+\bullet}$)
**55**	DihydroAsc Hex	$C_{21}H_{27}NO_{11}$	$[M-H]^-$, 15	**468** (20, $C_{21}H_{26}NO_{11}^-$), 288 (13, $C_{15}H_{14}NO_5^-$), 246 (36, $C_{13}H_{12}NO_4^-$), 226 (28, $C_{14}H_{12}NO_2^-$), 218 (17, $C_{12}H_{12}NO_3^-$), 216 (12, $C_{12}H_{10}NO_3^-$), 210 (23, $C_{13}H_8NO_2^-$), 204 (92, $C_{11}H_{10}NO_3^-$), 188 (100, $C_{11}H_{10}NO_2^-$), 179 (31, $C_6H_{11}O_6^-$), 172 (19, $C_{11}H_{10}NO^-$), 158 (14, $C_{11}H_8NO^-$), 116 (11, $C_8H_6N^-$), see reference [3]
**56**	$7MeSO-Heptyl-NH_2$	$C_8H_{19}NOS$	$[M+H]^+$, 20	**178** (52, $C_8H_{20}NOS^+$), 161 (26, $C_8H_{17}OS^+$), 160 (13, $C_8H_{18}NS^+$), 114 (100, $C_7H_{16}N^+$), 112 (9, $C_7H_{14}N^+$) 97 (20, $C_7H_{13}^+$), 95 (9, $C_7H_{11}^+$)
**57**	$8MeSO-Octyl-NH_2$	$C_9H_{21}NOS$	$[M+H]^+$, 20	**192** (100, $C_9H_{22}NOS^+$), 175 (15, $C_9H_{19}OS^+$), 174 (7, $C_9H_{20}NS^+$), 128 (100, $C_8H_{18}N^+$), 111 (5, $C_8H_{15}^+$), 109 (4, $C_8H_{13}^+$), 97 (5, $C_7H_{13}^+$)
**58**	Acetylagmatine	$C_7H_{16}N_4O$	$[M+H]^+$, 20	**173** (18, $C_7H_{17}N_4O^+$), 156 (26, $C_7H_{14}N_3O^+$), 131 (5, $C_6H_{15}N_2O^+$), 114 (100, $C_6H_{12}NO^+$), 113 (23, $C_6H_{13}N_2^+$), 72 (9, $C_4H_{10}N^+$)
**59**	Phenacetylagmatine	$C_{13}H_{20}N_4O$	$[M+H]^+$, 15	**249** (70, $C_{13}H_{21}N_4O^+$), 232 (40, $C_{13}H_{18}N_3O^+$), 207 (7, $C_{12}H_{19}N_2O^+$), 190 (100, $C_{12}H_{16}NO^+$), 189 (35, $C_{12}H_{17}N_2^+$), 120 (4, $C_8H_{10}N^+$), 114 (8, $C_5H_{12}N_2^+$)
**60**	Coumaroylagmatine	$C_{14}H_{20}N_4O_2$	$[M+H]^+$, 15	**277** (34, $C_{14}H_{21}N_4O_2^+$), 260 (21, $C_{14}H_{18}N_3O_2^+$), 218 (24, $C_{13}H_{16}NO_2^+$) 217 (20, $C_{13}H_{17}N_2O^+$), 147 (100, $C_9H_7O_2^+$), 131 (7, $C_5H_{15}N_4^+$), 119 (3, $C_8H_7O^+$), 114 (22, $C_5H_{12}N_3^+$)
**61**	Feruloylagmatine	$C_{15}H_{22}N_4O_3$	$[M+H]^+$, 15	**307** (74, $C_{15}H_{23}N_4O_3^+$), 290 (21, $C_{15}H_{20}N_3O_3^+$), 177 (100, $C_{10}H_9O_3^+$), 131 (9, $C_5H_{15}N_4^+$), 114 (10, $C_5H_{12}N_3^+$)
**62**	*N*-Phenacetyl-Asp	$C_{12}H_{13}NO_5$	$[M-H]^-$, 10	**250** (89, $C_{12}H_{12}NO_5^-$), 232 (3, $C_{12}H_{10}NO_4^-$), 206 (8, $C_{11}H_{12}NO_3^-$), 132 (100, $C_4H_6NO_4^-$), 115 (6, $C_4H_3O_4^-$), 114 (3, $C_4H_4NO_3^-$), 88 (7, $C_3H_6NO_2^-$)
**63**	SA	$C_7H_6O_3$	$[M-H]^-$, 15	identical CID mass spectrum as observed for authenticated standard
**64**	SGE	$C_{13}H_{16}O_8$	$[M-H]^-$, 15	**299** (1, $C_{13}H_{15}O_8^-$), 239 (1, $C_{11}H_{11}O_6^-$), 209 (2, $C_{10}H_9O_5^-$), 179 (16, $C_9H_7O_4^-$), 151 (8, $C_8H_7O_3^-$),137 (100, $C_7H_5O_3^-$), 93 (3, $C_6H_5O^-$)
**65**	SAG	$C_{13}H_{16}O_8$	$[M-H]^-$, 15	**299** (7, $C_{13}H_{15}O_8^-$), 137(100, $C_7H_5O_3^-$), 93(5, $C_6H_5O^-$)
**66**	2,5-DHBA Hex	$C_{13}H_{16}O_9$	$[M-H]^-$, 15	**315** (100, $C_{13}H_{15}O_9^-$), 153 (13, $C_7H_5O_4^-$), 152 (26, $C_7H_4O_4^{-\bullet}$), 109 (3, $C_6H_5O_2^-$), 108 (6, $C_6H_4O_2^{-\bullet}$)
**67**	2,5-DHBA Pent	$C_{12}H_{14}O_8$	$[M-H]^-$, 15	**285** (100, $C_{12}H_{13}O_8^-$), 153 (30, $C_7H_5O_4^-$), 152 (45, $C_7H_4O_4^{-\bullet}$), 109 (4, $C_6H_5O_2^-$), 108 (17, $C_6H_4O_2^{-\bullet}$)
**68**	2,3-DHBA 3-Xyl	$C_{12}H_{14}O_8$	$[M-H]^-$, 15	**285** (100, $C_{12}H_{13}O_8^-$), 153 (55, $C_7H_5O_4^-$), 152 (5, $C_7H_4O_4^{-\bullet}$), 109 (7, $C_6H_5O_2^-$)
**69**	9,12,13-Trihydroxy-10,15-octadecadienoic acid	$C_{18}H_{32}O_5$	$[M-H]^-$, 20	**327** (67, $C_{18}H_{31}O_5^-$), 309 (6, $C_{18}H_{29}O_4^-$), 291 (16, $C_{18}H_{27}O_3^-$), 239 (9, $C_{13}H_{19}O_4^-$), 229 (59, $C_{12}H_{21}O_4^-$), 221 (12, $C_{13}H_{17}O_3^-$), 211 (100, $C_{12}H_{19}O_3^-$), 185 (7, $C_{10}H_{17}O_3^-$), 183 (12, $C_{11}H_{19}O_2^-$), 177 (6, $C_{12}H_{17}O^-$), 171 (29, $C_9H_{15}O_3^-$)
**70**	oPDA	$C_{18}H_{28}O_3$	$[M-H]^-$, 25	**291** (56, $C_{18}H_{27}O_3^-$), 273 (23, $C_{18}H_{25}O_2^-$), 247 (59, $C_{17}H_{27}O^-$), 217 (10, $C_{15}H_{21}O^-$), 165 (100, $C_{11}H_{17}O^-$), 163 (21, $C_{11}H_{15}O^-$), 148 (21, $C_{10}H_{12}O^{-\bullet}$)
**71**	JA	$C_{12}H_{18}O_3$	$[M-H]^-$, 15	identical CID mass spectrum as observed for authenticated standard

**72**	JA Glc	$C_{18}H_{28}O_8$	$[M-H]^-$, 15	**317** (0, $C_{18}H_{27}O_8^-$), 209 (100, $C_{12}H_{17}O_3^-$)
**73**	Feruloylmalate (FerM)	$C_{14}H_{14}O_8$	$[M-H]^-$, 20	**309** (0, $C_{14}H_{13}O_8^-$), 193 (100, $C_{10}H_9O_4^-$), 178 (10, $C_9H_6O_4^{-\bullet}$), 149 (13, $C_9H_9O_2^-$), 134 (98, $C_8H_6O_2^{-\bullet}$), 133 (10, $C_4H_5O_5^-$)
**74**	G(8-*O*-4)FerM	$C_{24}H_{26}O_{12}$	$[M-H]^-$, 20	**505** (0, $C_{24}H_{25}O_{12}^-$), 389 (96, $C_{20}H_{21}O_8^-$), 341 (24, $C_{19}H_{17}O_6^-$), 195 (50, $C_{10}H_{11}O_4^-$), 193 (100, $C_{10}H_9O_4^-$), 165 (17, $C_9H_9O_3^-$)
**75**	S(8-*O*-4)FerM	$C_{25}H_{28}O_{13}$	$[M-H]^-$, 20	**535** (0, $C_{25}H_{27}O_{13}^-$), 419 (21, $C_{21}H_{23}O_9^-$), 371 (73, $C_{20}H_{19}O_7^-$), 225 (75, $C_{11}H_{13}O_5^-$), 195 (25, $C_{10}H_{11}O_{14}^-$), 193 (100, $C_{10}H_9O_4^-$)
**76**	G(8-5)FerM	$C_{24}H_{24}O_{11}$	$[M-H]^-$, 20	**487** (0, $C_{24}H_{23}O_{11}^-$), 371 (20, $C_{20}H_{19}O_7^-$), 353 (100, $C_{20}H_{17}O_6^-$), 343 (76, $C_{19}H_{17}O_6^-$), 338 (12, $C_{19}H_{14}O_6^{-\bullet}$), 322 (4, $C_{19}H_{14}O_5^{-\bullet}$), 309 (7, $C_{18}H_{13}O_5^-$), 294 (3, $C_{17}H_{10}O_5^{-\bullet}$), 181 (2, $C_9H_9O_4^-$), 133 (2, $C_4H_5O_5^-$), 115 (4, $C_4H_3O_4^-$)
**77**	5HO-FerM	$C_{14}H_{14}O_9$	in-source CID	325 (100, $C_{14}H_{13}O_9^-$), 209 (44, $C_{10}H_9O_5^-$), 133 (20, $C_4H_5O_5^-$)
**78**	Sinapoyl malate (SinM)	$C_{15}H_{16}O_9$	in-source CID	339 (69, $C_{15}H_{15}O_9^-$), 223 (100, $C_{11}H_{11}O_5^-$), 133 (7, $C_4H_5O_5^-$)
**79**	G(8-*O*-4)SinM	$C_{25}H_{28}O_{13}$	$[M-H]^-$, 20	**535** (0, $C_{25}H_{27}O_{13}^-$), 419 (24, $C_{21}H_{23}O_9^-$), 371 (14, $C_{20}H_{19}O_7^-$), 223 (100, $C_{11}H_{11}O_5^-$), 195 (58, $C_{10}H_{11}O_4^-$), 165 (20, $C_9H_9O_3^-$)
**80**	SinGlc	$C_{17}H_{22}O_{10}$	$[M-H]^-$, 15	**385** (18, $C_{17}H_{21}O_{10}^-$), 325 (6, $C_{15}H_{17}O_8^-$), 295 (10, $C_{14}H_{15}O_7^-$), 265 (8, $C_{13}H_{13}O_6^-$), 247 (12, $C_{13}H_{11}O_5^-$), 223 (34, $C_{11}H_{11}O_5^-$), 205(100, $C_{11}H_9O_4^-$), 190 ((4, $C_{10}H_6O_4^{-\bullet}$), 179 (2, $C_{10}H_{11}O_3^-$)
**81**	DiSinGlc	$C_{28}H_{32}O_{14}$	$[M-H]^-$, 20	**591** (15, $C_{28}H_{31}O_{14}^-$), 385 (7, $C_{17}H_{21}O_{10}^-$), 367 (19, $C_{17}H_{19}O_9^-$), 295(27, $C_{14}H_{15}O_7^-$), 277(17, $C_{14}H_{13}O_6^-$), 223(100, $C_{11}H_{11}O_5^-$), 205(99, $C_{11}H_9O_4^-$), 179 (7, $C_{10}H_{11}O_3^-$), 161 (11, $C_6H_9O_5^-$)
**82**	K 3-Rha-7-Rha	$C_{27}H_{30}O_{14}$	in-source CID	579 (100, $C_{27}H_{31}O_{14}^+$), 433 (14, $C_{21}H_{21}O_{10}^+$), 287 (2, $C_{15}H_{11}O_6^+$)
**83**	K 3-Glc-7-Rha	$C_{27}H_{30}O_{15}$	in-source CID	595 (100, $C_{27}H_{31}O_{15}^+$), 433 (9, $C_{21}H_{21}O_{10}^+$), 287 (2, $C_{15}H_{11}O_6^+$)
**84**	Q 3-Glc-7-Rha	$C_{27}H_{30}O_{16}$	$[M-H]^-$, 40	**609** (0, $C_{27}H_{29}O_{16}^-$), 446 (19, $C_{21}H_{18}O_{11}^{-\bullet}$), 301 (29, $C_{15}H_9O_7^-$), 300 (24, $C_{15}H_8O_7^{-\bullet}$), 299 (100, $C_{15}H_7O_7^-$), 271 (11, $C_{14}H_7O_6^-$)
**85**	K 3-(Rha-Glc)-7-Rha	$C_{33}H_{40}O_{19}$	$[M-H]^-$, 40	**739** (0, $C_{33}H_{39}O_{19}^-$), 593 (42, $C_{27}H_{29}O_{15}^-$), 430 (82, $C_{21}H_{18}O_{10}^{-\bullet}$), 285 (36, $C_{15}H_9O_6^-$), 284 (100, $C_{15}H_8O_6^{-\bullet}$), 283 (72, $C_{15}H_7O_6^-$)
**86**	Q 3-(DeoxyHex-Hex)-7-DeoxyHex	$C_{33}H_{40}O_{20}$	$[M-H]^-$, 40	**755** (3, $C_{33}H_{39}O_{20}^-$), 609 (8, $C_{27}H_{29}O_{16}^-$), 446 (100, $C_{21}H_{18}O_{11}^{-\bullet}$), 300 (24, $C_{15}H_8O_7^{-\bullet}$), 299 (53, $C_{15}H_7O_7^-$)
**87**	K 3-(Glc-Glc)-7-Rha	$C_{33}H_{40}O_{20}$	$[M-H]^-$, 40	**755** (0, $C_{33}H_{39}O_{20}^-$), 609 (6, $C_{27}H_{29}O_{16}^-$), 447 (18, $C_{21}H_{19}O_{11}^-$), 431 (18, $C_{21}H_{19}O_{10}^-$), 430 (9, $C_{21}H_{18}O_{10}^{-\bullet}$), 285 (100, $C_{15}H_9O_6^-$), 284 (13, $C_{15}H_8O_6^{-\bullet}$)
**88**	At-NCC-1	$C_{40}H_{48}N_4O_{13}$	$[M-H]^-$, 40	**791** (0, $C_{40}H_{47}N_4O_{13}^-$), 747 (58, $C_{40}H_{47}N_4O_{11}^-$), 624 (98, $C_{32}H_{38}N_3O_{10}^-$), 580 (85, $C_{31}H_{38}N_3O_8^-$), 400 (42, $C_{25}H_{26}N_3O_2^-$), 314 (25, $C_{14}H_{20}NO_7^-$), 287 (51, $C_{16}H_{19}N_2O_3^-$), 279 (100, $C_{17}H_{15}N_2O_2^-$), 253 (67, $C_{16}H_{17}N_2O^-$)
**89**	At-NCC-4	$C_{41}H_{50}N_4O_{13}$	$[M+H]^+$, 30	**807** (4, $C_{41}H_{51}N_4O_{13}^+$), 775 (25, $C_{40}H_{47}N_4O_{12}^+$), 684 (10, $C_{34}H_{42}N_3O_{12}^+$), 652 (29, $C_{33}H_{38}N_3O_{11}^+$), 645 (12, $C_{35}H_{41}N_4O_8^+$), 613 (100, $C_{34}H_{37}N_4O_7^+$), 522 (17, $C_{28}H_{32}N_3O_4^+$), 490 (50, $C_{27}H_{28}N_3O_6^+$), 460 (18, $C_{26}H_{26}N_3O_5^+$), 448 (17, $C_{25}H_{26}N_3O_5^+$), 357 (15, $C_{19}H_{21}N_2O_5^+$), 337 (11, $C_{19}H_{17}N_2O_4^+$), 325 (9, $C_{18}H_{17}N_2O_4^+$), 204 (7, $C_{11}H_{10}NO_3^+$), 166 (4, $C_9H_{12}NO_2^+$)
**90**[b]	UNK-1 (indolic)	$C_{11}H_{10}N_2O$	$[M+H]^+$, 15	**187** (100, $C_{11}H_{11}N_2O^+$), 144 (77, $C_9H_6NO^+$), see reference [2]
**91**	UNK-2 (indolic)	$C_{12}H_{12}N_2O_4$	$[M-H]^-$, 15	**247** (1, $C_{12}H_{11}N_2O_4^-$), 160 (100, $C_9H_6NO_2^-$), 132 (4, $C_8H_6NO^-$), see reference [3]
**92**	UNK-3 (Hexoside, indolic aglycone $C_9H_8N_2O_2$)	$C_{15}H_{18}N_2O_7$	$[M-H]^-$, 15	**337** (100, $C_{15}H_{17}N_2O_7^-$), 217 (32, $C_{11}H_9N_2O_3^-$), 175 (84, $C_9H_7N_2O_2^-$), 147 (55, $C_8H_7N_2O^-$).
**93**	UNK-4 (Hexoside, indolic aglycone $C_9H_7NO_3$)	$C_{15}H_{17}NO_8$	$[M-H]^-$, 15	**338** (19, $C_{15}H_{16}NO_8^-$), 176 (100, $C_9H_6NO_3^-$), 132 (8, $C_8H_6NO^-$) see reference [3]
**94**	UNK-5 (Hexoside, indolic aglycone $C_{10}H_9NO_3$)	$C_{16}H_{19}NO_8$	$[M-H]^-$, 15	**352** (15, $C_{16}H_{18}NO_8^-$), 190 (100, $C_{10}H_8NO_3^-$), 146 (11, $C_9H_8NO^-$)
**95**	UNK-6	$C_{10}H_9NO_3$	$[M+H]^+$, 20	**192** (1, $C_{10}H_{10}NO_3^+$), 178 (3, $C_9H_8NO_3^+$), 160 (100, $C_9H_6NO_2^+$), 132 (16, $C_8H_6NO^+$), 104 (12, $C_7H_6N^+$)
**96**	UNK-7	$C_{10}H_9NO_3$	$[M+H]^+$, 20	**192** (3, $C_{10}H_{10}NO_3^+$), 174 (100, $C_{10}H_8NO_2^+$), 148 (3, $C_9H_{10}NO^+$), 146 (5, $C_9H_8NO^+$), 130 (3, $C_9H_8N^+$), 118 (30, $C_8H_8N^+$)
**97**	UNK-8	$C_{11}H_{11}NO_3$	$[M+H]^+$, 20	**206** (2, $C_{11}H_{12}NO_3^+$), 192 (10, $C_{10}H_{10}NO_3^+$), 174 (100, $C_{10}H_8NO_2^+$), 147 (9, $C_9H_9NO^{+\bullet}$), 146 (5, $C_9H_8NO^+$), 130 (3, $C_9H_8NO^+$), 118 (15, $C_8H_8N^+$)
**98**	UNK-9 (Hydroxycamalexin-#3)	$C_{11}H_8N_2OS$	$[M+H]^+$, 25	**217** (89, $C_{11}H_9N_2OS^+$), 199 (10, $C_{11}H_7N_2S^+$), 198 (5, $C_{11}H_7N_2S^{+\bullet}$), 189 (100, $C_{10}H_9N_2S^+$), 184 (2, $C_{11}H_8N_2O^{+\bullet}$), 176 (12, $C_9H_6NOS^+$), 173 (13, $C_{10}H_7NS^{+\bullet}$), 172 (32, $C_{10}H_6NS^+$), 162 (37, $C_9H_8NS^+$), 158 (21, $C_9H_6N_2O^{+\bullet}$), 156 (65, $C_{10}H_8N_2^{+\bullet}$), 155 (35, $C_{10}H_7N_2^+$), 148 (3, $C_8H_6NS^+$), 132 (6, $C_8H_6NO^+$), 118 (3, $C_8H_8N^+$), 117 (4, $C_8H_7N^{+\bullet}$), 104 (11, $C_7H_6N^+$)
**99**	UNK-10 (Hexoside, aglycone $C_6H_{11}NO_3$)	$C_{12}H_{21}NO_8$	$[M+H]^+$, 10	**308** (24, $C_{12}H_{22}NO_8^+$), 146 (100, $C_6H_{12}NO_3^+$), 128 (6, $C_6H_{10}NO_2^+$), 100 (2, $C_5H_{10}NO^+$)
**100**	UNK-11 (Malate ester)	$C_{16}H_{20}O_8$	$[M-H]^-$, 10	**339** (14, $C_{16}H_{19}O_8^-$), 223 (99, $C_{12}H_{15}O_4^-$), 179 (100, $C_{11}H_{15}O_2^-$), 135 (5, $C_{10}H_{15}^-$), 133 (11, $C_4H_5O_5^-$), 115 (4, $C_4H_3O_4^-$)
**101**	UNK-12	$C_{10}H_{20}N_2O_3S$	$[M+H]^+$, 10	**249** (100, $C_{10}H_{21}N_2O_3S^+$), 232 (55, $C_{10}H_{18}NO_3S^+$), 186 (29, $C_9H_{16}NOS^+$), 130 (3, $C_5H_8NO_3^+$), 120 (9, $C_5H_{14}NS^+$), 103 (55, $C_5H_{11}S^+$)
**102**	UNK-13	$C_{13}H_{10}N_2O_4S$	$[M+H]^+$, 20	**291** (0, $C_{13}H_{11}N_2O_4S^+$), 160 (100, $C_9H_6NO_2^+$), 132 (7, $C_4H_6NO_2S^+$), 86 (2, $C_3H_4NS^+$)
**103**	UNK-14	$C_{12}H_{19}N_3O_5$	$[M-H]^-$, 15	**284** (65, $C_{12}H_{18}N_3O_5^-$), 266 (50, $C_{12}H_{16}N_3O_4^-$), 242 (9, $C_{10}H_{16}N_3O_4^-$), 240 (4, $C_{11}H_{18}N_3O_3^-$), 224 (14, $C_{10}H_{14}N_3O_3^-$), 198 (100, $C_9H_{16}N_3O_2^-$), 180 (25, $C_9H_{14}N_3O^-$), 145 (5, $C_5H_9N_2O_3^-$), 127 (6, $C_5H_7N_2O_2^-$)
**104**	UNK-15	$C_{17}H_{17}N_3O_5S_2$	$[M+H]^+$, 10	**408** (46, $C_{17}H_{18}N_3O_5S_2^+$), 201 (100, $C_{11}H_9N_2S^+$)

**105**	UNK-16	$C_{15}H_{13}N_3O_5S$	$[M-H]^-$, 10	**346** (100, $C_{15}H_{12}N_3O_5S^-$), 312 (13, $C_{15}H_{10}N_3O_5^-$), 271 (24, $C_{13}H_7N_2O_3S^-$), 268 (5, $C_{14}H_{10}N_3O_3^-$), 213 (5, $C_7H_5N_2O_4S^-$), 187 (67, $C_6H_7N_2O_3S^-$), 169 (6, $C_6H_5N_2O_2S^-$)
**106**	UNK-17	$C_{15}H_{16}N_2O_6$	$[M-H]^-$, 20	**319** (25, $C_{15}H_{15}N_2O_6^-$), 229 (1, $C_{12}H_9N_2O_3^-$), 199 (4, $C_{11}H_7N_2O_2^-$), 156 (100, $C_6H_6NO_4^-$), 128 (1, $C_5H_6NO_3^-$)
**107**	UNK-18	$C_{12}H_{20}N_2O_4$	$[M-H]^-$, 15	**255** (100, $C_{12}H_{19}N_2O_4^-$), 213 (55, $C_{10}H_{17}N_2O_3^-$), 169 (77, $C_9H_{17}N_2O^-$), 140 (4, $C_7H_{10}NO_2^-$), 116 (8, $C_5H_{10}NO_2^-$)
**108**	UNK-19	$C_{17}H_{22}N_2O_7$	$[M-H]^-$, 15	**365** (63, $C_{17}H_{21}N_2O_7^-$), 248 (30, $C_9H_{14}NO_7^-$), 203 (10, $C_{11}H_{11}N_2O_2^-$), 185 (100, $C_{11}H_9N_2O^-$), 179 (9, $C_6H_{11}O_6^-$), 161 (8, $C_6H_9O_5^-$), 155 (9, $C_{10}H_7N_2^-$), 116 (95, $C_8H_6N^-$)
**109**	UNK-20 ($C_{11}H_{20}O_3$, sulfated)	$C_{11}H_{20}O_6S$	$[M-H]^-$, 20	**279** (44, $C_{11}H_{19}O_6S^-$), 199 (56, $C_{11}H_{19}O_3^-$), 97 (100, HSO_4^-)
**110**	UNK-21	$C_{16}H_{19}NO_9$	$[M-H]^-$, 30	**368** (0, $C_{16}H_{18}NO_9^-$), 206 (51, $C_{10}H_8NO_4^-$), 205 (100, $C_{10}H_7NO_4^{-\bullet}$), 190 (21, $C_9H_4NO_4^-$), 162 (66, $C_9H_8NO_2^-$), 147 (77, $C_8H_5NO_2^{-\bullet}$), 146 (72, $C_8H_4NO_2^-$)
**111**	UNK-22 (Dihexoside, aglycone $C_9H_7NO_2$)	$C_{21}H_{27}NO_{12}$	$[M-H]^-$, 20	**484** (93, $C_{21}H_{26}NO_{12}^-$), 341 (100, $C_{12}H_{21}O_{11}^-$), 322 (4, $C_{15}H_{16}NO_7^-$), 304 (63, $C_{15}H_{14}NO_6^-$), 214 (3, $C_{12}H_8NO_3^-$), 179 (18, $C_6H_{11}O_6^-$), 161 (9, $C_6H_9O_5^-$), 160 (17, $C_9H_6NO_2^-$)
**112**	UNK-23 (Dihexoside, aglycone $C_9H_7NO_2$)	$C_{21}H_{27}NO_{12}$	$[M-H]^-$, 20	**484** (36, $C_{21}H_{26}NO_{12}^-$), 341 (30, $C_{12}H_{21}O_{11}^-$), 322 (4, $C_{15}H_{16}NO_7^-$), 304 (100, $C_{15}H_{14}NO_6^-$), 262 (5, $C_{13}H_{12}NO_5^-$), 214 (6, $C_{12}H_8NO_3^-$), 179 (7, $C_6H_{11}O_6^-$), 161 (6, $C_6H_9O_5^-$), 160 (7, $C_9H_6NO_2^-$)
**113**	UNK-24 (Dihexoside, aglycone $C_9H_7NO_3$)	$C_{21}H_{27}NO_{13}$	$[M-H]^-$, 20	**500** (43, $C_{21}H_{26}NO_{13}^-$), 341 (4, $C_{12}H_{21}O_{11}^-$), 320 (7, $C_{15}H_{14}NO_7^-$), 176 (100, $C_9H_6NO_3^-$), 158 (30, $C_9H_4NO_2^-$), 132 (1, $C_8H_6NO^-$)

[a] elemental composition of fragment ions supported by accurate mass measurements (±10 ppm)

References: [1] Rochfort et al. *Phytochemistry* **2008**, *59*, 1671; [2] Böttcher et al. *Plant Cell* **2009**, *21*, 1830; [3] Böttcher et al. *Plant Phys.* **2014**, *165*, 841.

[b] UNK = unknown compound or only putatively characterised (compound 90-113).
